# YlAaf1-YlAaf2, a bipartite SANT domain-containing complex of transcriptional activator, promotes filamentous growth in the dimorphic yeast *Yarrowia lipolytica*

**DOI:** 10.1128/msphere.00403-25

**Published:** 2025-08-18

**Authors:** Meng-Yang Xu, Zhen-Hua Wang, Xiang-Dong Gao

**Affiliations:** 1 Hubei Key Laboratory of Cell Homeostasis, College of Life Sciences, Wuhan University 98436, Wuhan, China; 2 Division of Natural and Applied Sciences, Duke Kunshan University 517759, Kunshan, China; University of Michigan Michigan Medicine, Ann Arbor, Michigan, USA

**Keywords:** transcription factor, filamentous growth, filamentation, dimorphic transition, dimorphism

## Abstract

The yeast-to-filament transition is important for dimorphic fungi to adapt to different environmental conditions. Transcriptional activators that activate downstream target genes in response to environmental stimulations are particularly important for the control of this morphological transition. However, only a limited number of key activators of filamentation have been identified. Here, we report YlAaf1 and YlAaf2, a novel pair of SANT domain-containing transcription factors that promote filamentation in the dimorphic yeast *Yarrowia lipolytica*. We show that both deletion and co-overexpression of Yl*AAF1* and Yl*AAF2* affect filamentation substantially. We also show that YlAaf1 and YlAaf2 interact with each other physically, and the bipartite complex has transcriptional activation activity. In addition, we show that YlAaf1 and YlAaf2 respond to alkaline pH and are upregulated by YlRim101 and Mhy1, two key transcription factors important for alkaline-induced filamentation. Lastly, we show that YlAaf1-YlAaf2 and Mhy1 are direct regulatory targets of each other.

**IMPORTANCE:**

Transcription factors play critical roles in the control of the yeast-to-filament transition in dimorphic fungi. However, many of them contain zinc finger or zinc cluster DNA-binding domains, but not SANT domain, the latter of which is initially identified in nuclear receptor co-repressors and the subunits of many chromatin-remodeling complexes. In this paper, we report two novel SANT domain-containing transcription factors, YlAaf1 and YlAaf2, that promote filamentation in *Yarrowia lipolytica*. YlAaf1 and YlAaf2 are closely related to *Candida albicans* Aaf1, which also appears to promote filamentation. YlAaf1 and YlAaf2 are unique in that they are not simply redundant in function but instead form a bipartite transcriptional activator. One subunit, YlAaf2, is highly responsive to environmental stimulation such as alkaline pH, a strong inducer of the yeast-to-filament transition. We identified a crosstalk between YlAaf1-YlAaf2 and Mhy1, the master regulator of filamentation. Our results provide new insights into the regulatory networks that control the yeast-to-filament transition in *Y. lipolytica*.

## INTRODUCTION

Many fungal species can grow in the oval-shaped yeast form or in filamentous forms such as hyphae and pseudohyphae. The yeast-to-filament transition can be induced by environmental factors such as nutrient starvation, alkaline pH, serum, and low levels of oxygen (1, 2). This dimorphic switch allows fungal cells to adapt to new environments (2). In some pathogenic fungi, including the opportunistic human pathogen *Candida albicans*, the morphological switch is essential for infection (3, 4). It is thus important to understand the regulatory mechanisms that govern this morphological switch in different fungal species.

*Yarrowia lipolytica* is a non-conventional yeast that has industrial applications in the production of organic acids, high-value proteins, biofuels, and other products (5, 6). Like *C. albicans*, *Y. lipolytica* also undergoes dimorphic transition between the yeast form and filamentous forms in response to environmental stimulations, including poor nutrients, alkaline pH, or low oxygen (7, 8). During industrial fermentation, *Y. lipolytica* cells often suffer from various stresses and, as a result, switch to filamentous forms, which are often unfavorable for industrial fermentation (9). This has been reported to reduce the yield of fermentation products (10). This problem can be circumvented by utilizing mutants that are locked in the yeast form or, in another scenario, mutants that are locked in certain types of filamentous forms (10, 11).

Previous studies have identified several transcription factors that control the dimorphic transition in *Y. lipolytica*. Some of them positively regulate the yeast-to-filament transition, such as Hoy1, Mhy1, YlRim101, and Mar1 (12–15). Others negatively regulate this process, such as YlTec1, Znc1, Fts1, and Fts2 (16–19). Among the positive regulators, the C_2_H_2_-type zinc finger protein Mhy1 plays a key role in the control of the yeast-to-filament transition. It appears to be the regulatory target of various signaling pathways, including the nutrient-sensing TORC1-Sch9 signaling pathway and the pH-sensing Rim101 pathway (14, 20). It is also regulated by transcriptional regulators Fts1, Fts2, Mar1, and the general transcriptional repressor YlTup1-YlSsn6 (15, 18, 19).

The SANT domain was initially identified in nuclear receptor co-repressors and the subunits of many chromatin-remodeling complexes (21, 22). It was named after Swi3 (switching-defective protein 3), Ada2 (adaptor 2), N-CoR (nuclear receptor co-repressor), and TFIIIB (transcription factor). The SANT domain was structurally similar to the DNA-binding domain of Myb-related proteins. Both are helix-loop-helix motifs composed of three *α*-helices that are functionally diverse and capable of interacting with DNA and proteins (23, 24). *C. albicans* Aaf1 (adhesion and aggregation factor 1) contains two tandem SANT domains. It was initially identified as a protein that affects adherence as it increased cell adherence and flocculence when ectopically expressed in *Saccharomyces cerevisiae* (25–27). It was later found to regulate filamentation as *C. albicans aaf1*Δ cells exhibited reduced colony wrinkling but increased invasive growth on Spider medium at 37°C (28). In addition, CaAaf1 caused invasive growth when overexpressed in *S. cerevisiae* haploid *flo8*Δ and *flo11*Δ mutants, which are defective in invasive growth (27). CaAaf1 also affects resistance to antifungal drugs, as *aaf1*Δ cells exhibited increased resistance to fluconazole (28).

In this study, we report the identification of YlAaf1-YlAaf2, a novel bipartite transcriptional activator that promotes filamentous growth in *Y. lipolytica*. The two proteins are closely related to *C. albicans* Aaf1 and contain two tandem SANT domains. We examined the functional and physical interactions between YlAaf1 and YlAaf2. We also investigated how they respond to alkaline pH and how they control filamentation.

## RESULTS

### Identification of YlAaf1 and YlAaf2, a pair of interdependent activators of filamentous growth in *Y. lipolytica*

**Fig 1:**
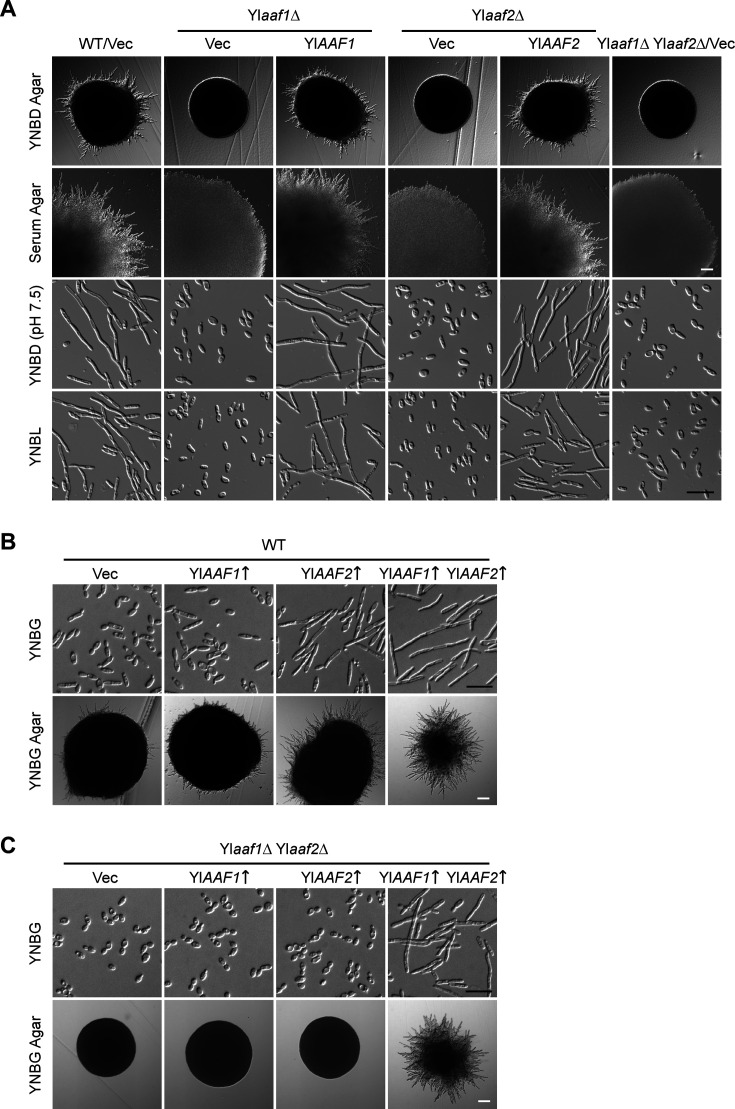
Phenotypes of Yl*aaf1*Δ, Yl*aaf2*Δ, and Yl*aaf1*Δ Yl*aaf2*Δ cells and cells overexpressing Yl*AAF1*, Yl*AAF2*, or Yl*AAF1*-Yl*AAF2*. (**A**) Deletion of Yl*AAF1* or Yl*AAF2* severely impaired filamentation. Cells of wild-type strain carrying plasmid pINA445 (WT/Vec), Yl*aaf1*Δ carrying pINA445 (Yl*aaf1*Δ/Vec) or pINA445-P_5955 bp_-YlAAF1 (Yl*aaf1*Δ/Yl*AAF1*), Yl*aaf2*Δ carrying pINA445 (Yl*aaf2*Δ/Vec) or pINA445-P_3545 bp_-YlAAF2 (Yl*aaf2*Δ/Yl*AAF2*), and Yl*aaf1*Δ Yl*aaf2*Δ carrying pINA445 (Yl*aaf1*Δ Yl*aaf2*Δ/Vec) were grown at 30°C for 36 h on yeast nitrogen base-dextrose (YNBD) (glucose-containing) agar, 60 h on serum agar, 16 h in liquid YNBD (pH 7.5) medium, and 24 h in liquid yeast nitrogen base-lactate (YNBL) (lactate-containing) medium. (**B**) Yl*AAF2* overexpression enhanced filamentation, and Yl*AAF1*-Yl*AAF2* co-overexpression caused strong filamentation in wild-type cells. Cells of wild-type strain carrying plasmid pYL13 (Vec), pYL13-YlAAF1 (Yl*AAF1*↑), pYL13-YlAAF2 (Yl*AAF2*↑), or pYL13-YlAAF1-P_YlTEF1_-YlAAF2 (Yl*AAF1*↑ Yl*AAF2*↑) were grown at 30°C for 16 h in liquid yeast nitrogen base-glycerol (YNBG) (glycerol-containing) medium and 36 h on YNBG agar. (**C**) Yl*AAF1* and Yl*AAF2* are interdependent for function in filamentation. Yl*aaf1*Δ Yl*aaf2*Δ cells carrying plasmid pYL13 (Vec), pYL13-YlAAF1 (Yl*AAF1*↑), pYL13-YlAAF2 (Yl*AAF2*↑), or pYL13-YlAAF1-P_YlTEF1_-YlAAF2 (Yl*AAF1*↑ Yl*AAF2*↑) were grown at 30°C for 16 h in liquid YNBG medium and 36 h on YNBG agar. White scale bars, 100 µm. Black scale bars, 20 µm.

By zeta-*URA3* insertional mutagenesis of the *Y. lipolytica* genome, which randomly inserts zeta-*URA3* into the chromosome and generates mutations (18, 29), a mutant of *YALI0E23925g* that exhibited no filamentous growth on filament-inducing media was isolated. Subsequent deletion of this gene yielded an identical phenotype (see below and Fig. 1A). This finding suggests that *YALI0E23925g* is important for filamentation. The encoded protein YALI0E23925p is closely related to *C. albicans* Aaf1, a regulatory protein that also appears to promote filamentation (28). *Y. lipolytica* genome also encodes another Aaf1-related protein, YALI0E06105p. YALI0E23925p and YALI0E06105p share 43.2% and 33.6% amino acid sequence identity with CaAaf1, respectively. We thus named them YlAaf1 (YALI0E23925p) and YlAaf2 (YALI0E06105p).

To evaluate the functions of YlAaf1 and YlAaf2 in filamentous growth, their encoding genes were deleted in the wild-type strain PO1a. Compared to the wild-type strain, all the Yl*aaf1*Δ and Yl*aaf2*Δ single mutants and the Yl*aaf1*Δ Yl*aaf2*Δ double mutant were severely impaired in filamentous growth under several filament-inducing conditions. On yeast nitrogen base-dextrose (YNBD) agar, wild-type cells formed colonies with many radial filaments at the edge. In contrast, cells of all three mutants formed smooth colonies devoid of any filaments (Fig. 1A, row 1). On serum agar, a nutrient-deficient medium that stimulates much stronger filamentation than YNBD agar, wild-type cells formed colonies with long radial filaments. In contrast, cells of all three mutants formed colonies with very short radial filaments (Fig. 1A, row 2). These mutants also exhibited severe defects in filamentation in liquid media. In both filament-inducing YNBD (pH 7.5) and yeast nitrogen base-lactate (YNBL) (lactate-containing) media, wild-type cells formed long filaments. In contrast, cells of all three mutants did not form filaments. They were either oval-shaped or slightly elongated (Fig. 1A, rows 3 and 4). Introduction of wild-type Yl*AAF1* and Yl*AAF2* genes restored filamentation to Yl*aaf1*Δ and Yl*aaf2*Δ cells, respectively. This result suggests that both YlAaf1 and YlAaf2 are essential for filamentous growth in *Y. lipolytica*.

To explore whether increased YlAaf1 or YlAaf2 expression may enhance filamentous growth, the Yl*AAF1* and Yl*AAF2* genes were overexpressed in wild-type cells under the control of the strong Yl*TEF1* promoter, and cells were examined for filamentous growth under conditions that do not favor filamentation. Wild-type cells were oval-shaped or mildly elongated in liquid yeast nitrogen base-glycerol (YNBG) medium and formed colonies with few radial filaments on YNBG agar (Fig. 1B, column 1). Cells overexpressing Yl*AAF1* exhibited almost the same phenotype as wild-type cells. However, cells overexpressing Yl*AAF2* formed filaments in liquid YNBG medium and formed colonies with longer radial filaments than wild-type cells on YNBG agar (Fig. 1B, columns 2 and 3). Remarkably, cells co-overexpressing Yl*AAF1* and Yl*AAF2* formed long filaments in liquid YNBG medium and formed colonies with long radial filaments on YNBG agar (Fig. 1B, column 4). This phenotype is stronger than that of overexpressing Yl*AAF2* alone. This result suggests that YlAaf1 and YlAaf2 are activators of filamentation, and they may depend on each other for function.

To examine whether YlAaf1 and YlAaf2 may work together to function, the Yl*AAF1* and Yl*AAF2* genes were overexpressed individually in a Yl*aaf1*Δ Yl*aaf2*Δ strain, and the cells were examined under non-filament-inducing conditions. In both cases, no filamentation was observed when the cells were grown either in liquid YNBG medium or on YNBG agar. Only when Yl*AAF1* and Yl*AAF2* were co-overexpressed, strong filamentation was observed (Fig. 1C). This result suggests that YlAaf1 and YlAaf2 depend on each other for function in filamentation.

Together, our results suggest that YlAaf1 and YlAaf2 are a pair of novel activators of filamentous growth in *Y. lipolytica*. The two proteins are interdependent for function.

### YlAaf1 and YlAaf2 are putative transcription factors that each contain two SANT domains

**Fig 2:**
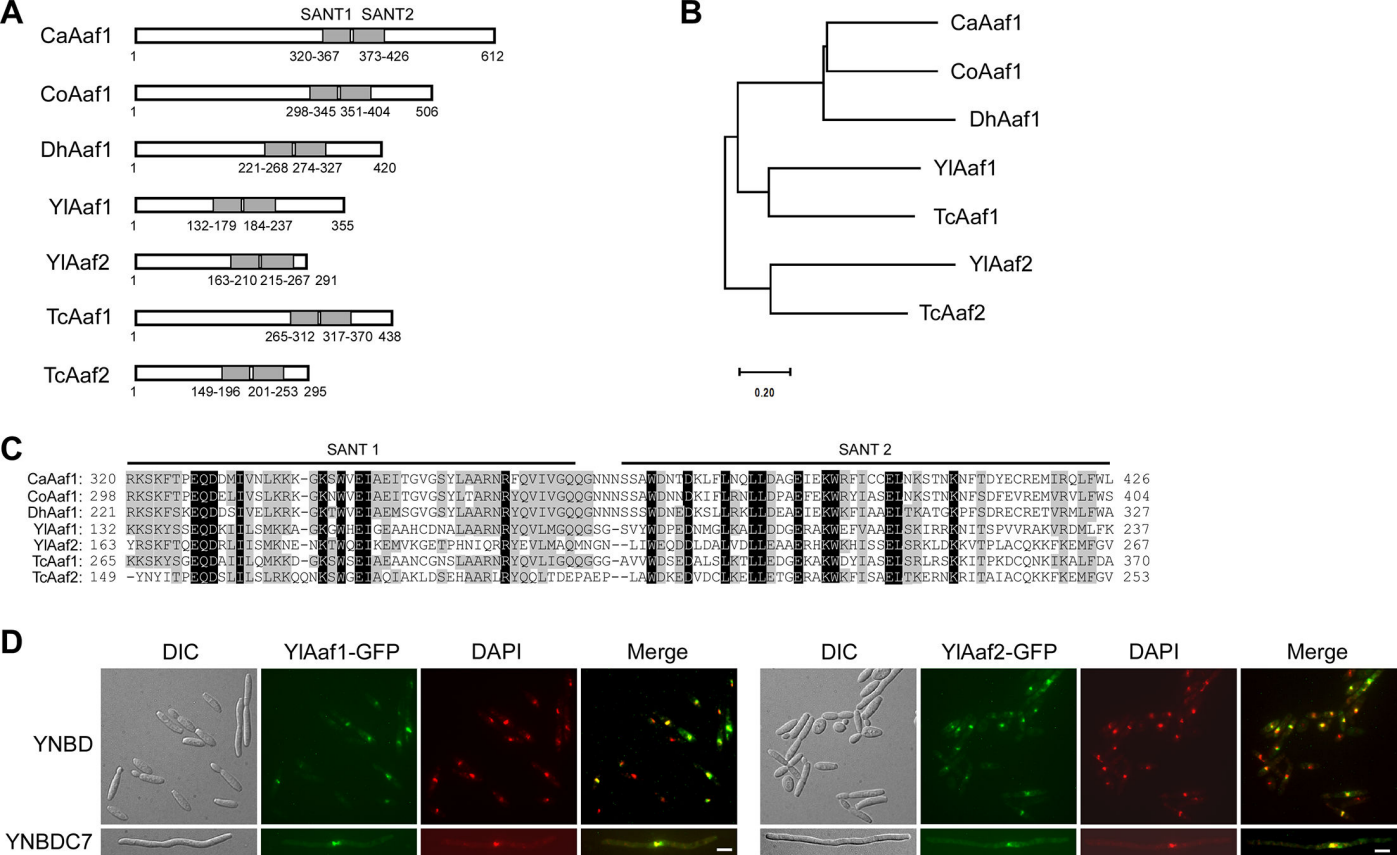
YlAaf1 and YlAaf2 are putative transcription factors that each contain two SANT domains. (**A**) Schematic representation of domain organization in *Candida albicans* CaAaf1, *Candida orthopsilosis* CoAaf1 (CORT_0C03600), *Y. lipolytica* YlAaf1 (YALI0E23925p) and YlAaf2 (YALI0E06105p), *Trichomonascus ciferrii* TcAaf1 (A0A642V0Z3) and TcAaf2 (A0A642UZ06), and *Debaromyces hansenii* DhAaf1 (DEHA2F17182p). The two SANT domains are depicted. (**B**) Phylogenetic tree of the seven Aaf1-related proteins. The neighbor-joining tree was constructed using MEGA 11. (**C**) Sequence alignment of the two SANT domains of the seven Aaf1-related proteins. Identical and similar residues are highlighted in dark and gray shades, respectively. (**D**) YlAaf1-GFP and YlAaf2-GFP localize to the nucleus. Yl*aaf1*Δ cells carrying plasmid pYL14-YlAAF1 and Yl*aaf2*Δ cells carrying pYL14-YlAAF2 were grown at 30°C for 16 h in liquid YNBD and YNBDC7 media. The nucleus was visualized by 4′,6′-diamidino-2-phenylindole (DAPI) staining. Bar, 5 µm.

YlAaf1 (355 a.a.) and YlAaf2 (291 a.a.) share 35.9% amino acid sequence identity with each other. They both contain two tandem SANT domains (Fig. 2A), which are structurally related to the DNA-binding domain of Myb-related transcription factors. Proteins homologous to YlAaf1 and YlAaf2 are widespread in yeasts. The closest homologs are TcAaf1 (A0A642V0Z3) and TcAaf2 (A0A642UZ06), respectively, in *Trichomonascus ciferrii* (Fig. 2A and B). The regions that share the highest amino acid sequence identity among these proteins are the two tandem SANT domains (Fig. 2C). Consistent with a putative role as transcription factor, GFP-tagged YlAaf1 and YlAaf2 localized to the nucleus in *Y. lipolytica* cells (Fig. 2D). Thus, YlAaf1 and YlAaf2 may activate filamentation by regulating gene expression.

### YlAaf1 and YlAaf2 physically interact with each other

**Fig 3:**
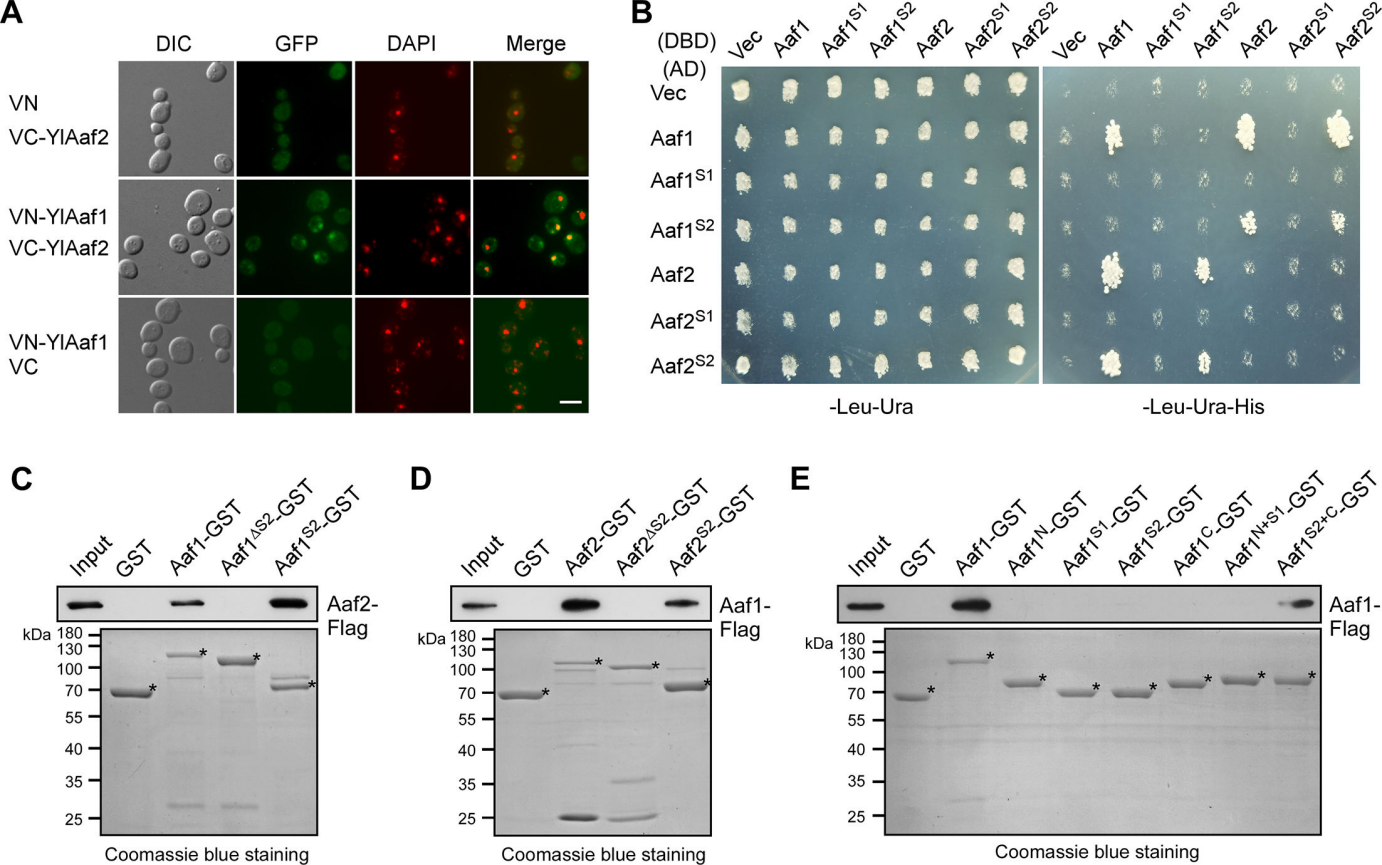
YlAaf1 and YlAaf2 physically interact with each other. (**A**) YlAaf1 interacts with YlAaf2 in the BiFC assay in *S. cerevisiae*. Cells of strain YEF473A carrying plasmid pairs pVN1/pVC1-YlAAF2, pVN1-YlAAF1/pVC1-YlAAF2, or pVN1-YlAAF1/pVC1 were grown at 30°C for 16 h in liquid SC-Ura-His medium. VN and VC represent the N-terminal and C-terminal regions of Venus (a GFP variant), respectively. Green fluorescence was examined by fluorescence microscopy. The nucleus was visualized by DAPI staining. Bar, 5 µm. (**B**) YlAaf1 and YlAaf2 interact via their SANT2 domains in a yeast two-hybrid assay. Cells of haploid *S. cerevisiae* strain pJ69-4A carrying pGAD-C1 (AD, vector), pGAD-YlAAF1 segments, or pGAD-YlAAF2 segments were mated with cells of haploid strain pJ69-4α carrying pGBDU-C1 (DBD, vector), pGBDU-YlAAF1 segments, or pGBDU-YlAAF2 segments. The resulting diploid cells grown on SC-Leu-Ura agar were transferred to SC-Leu-Ura-His agar through replica plating and grown at 30°C for 3 days. The full-length YlAaf1, YlAaf1^S1^ (a.a. 126-185, SANT1 domain), YlAaf1^S2^ (a.a. 178-243, SANT2 domain), full-length YlAaf2, YlAaf2^S1^ (a.a. 157-216, SANT1 domain), and YlAaf2^S2^ (a.a. 207-277, SANT2 domain) were examined. Cell growth on SC-Leu-Ura-His agar indicates protein interaction. (**C**) Bacterially expressed YlAaf1-SANT2 directly interacts with YlAaf2 in the GST pull-down assay. MBP-GST, MBP-YlAaf1-GST, MBP-YlAaf1 segments-GST, and MBP-YlAaf2-Flag produced in *Escherichia coli* cells were used. The full-length YlAaf1, YlAaf1^ΔS2^ (lacking a.a. 184-237 containing SANT2 domain), and YlAaf1^S2^ (a.a. 178-243, SANT2 domain) were examined for interaction. (**D**) Bacterially expressed YlAaf2-SANT2 directly interacts with YlAaf1 in the GST pull-down assay. The full-length YlAaf2, YlAaf2^ΔS2^ (lacking a.a. 213-267 containing SANT2 domain), and YlAaf2^S2^ (a.a. 207-277, SANT2 domain) were examined for interaction. (**E**) Homotypic interaction between bacterially expressed YlAaf1 is mediated by SANT2 and the C-terminal region. The full-length YlAaf1, YlAaf1^N^ (a.a. 1-125, N-terminal region), YlAaf1^S1^ (a.a. 126-185, SANT1 domain), YlAaf1^S2^ (a.a. 178-243, SANT2 domain), YlAaf1^C^ (a.a. 244-355, C-terminal region), YlAaf1^N+S1^ (a.a. 1-185), and YlAaf1^S2+C^ (a.a. 178-355) were examined for interaction.

Since YlAaf1 and YlAaf2 are interdependent for filamentation, we asked whether they may interact with each other and function in a complex. By using bimolecular fluorescence complementation (BiFC) assay in the budding yeast *Saccharomyces cerevisiae*, a physical interaction between YlAaf1 and YlAaf2 was detected in the nucleus (Fig. 3A). Seventeen percent of cells expressing VN-YlAaf1/VC-YlAaf2 (*n* > 200) exhibited the GFP signal in the nucleus (VN and VC denote the N-terminal and C-terminal regions of Venus, a GFP variant, respectively), whereas none of the cells expressing VN/VC-YlAaf2 or VN-YlAaf1/VC (*n* > 200) exhibited the GFP signal in the nucleus. This interaction was also detected in yeast two-hybrid assay, and the interaction appears to be mediated by the SANT2 domains of YlAaf1 and YlAaf2 (Fig. 3B). Interestingly, YlAaf1 also interacted with other YlAaf1, but none of its two SANT domains is sufficient to mediate this interaction (Fig. 3B).

To examine whether YlAaf1 may interact directly with YlAaf2, the two proteins and their truncated segments were tagged with GST or Flag at their C-terminus and expressed in *Escherichia coli* cells. To increase protein solubility, a maltose-binding protein (MBP) tag was added to these proteins at their N-terminus. GST pull-down assay confirmed that YlAaf1 and YlAaf2 interact directly with each other, and this interaction is mediated by the SANT2 domains of YlAaf1 and YlAaf2 (Fig. 3C and D). In addition, this assay showed that YlAaf1 interacted with other YlAaf1, and this interaction is mediated by the region that contains SANT2 and the C-terminal region (Fig. 3E). Neither the SANT2 domain nor the C-terminal region alone is sufficient to mediate this interaction.

Together, our results indicate that YlAaf1 physically interacts with YlAaf2 and other YlAaf1. The SANT2 domain is important for these interactions.

### The YlAaf1-YlAaf2 complex has transcriptional activation activity

**Fig 4:**
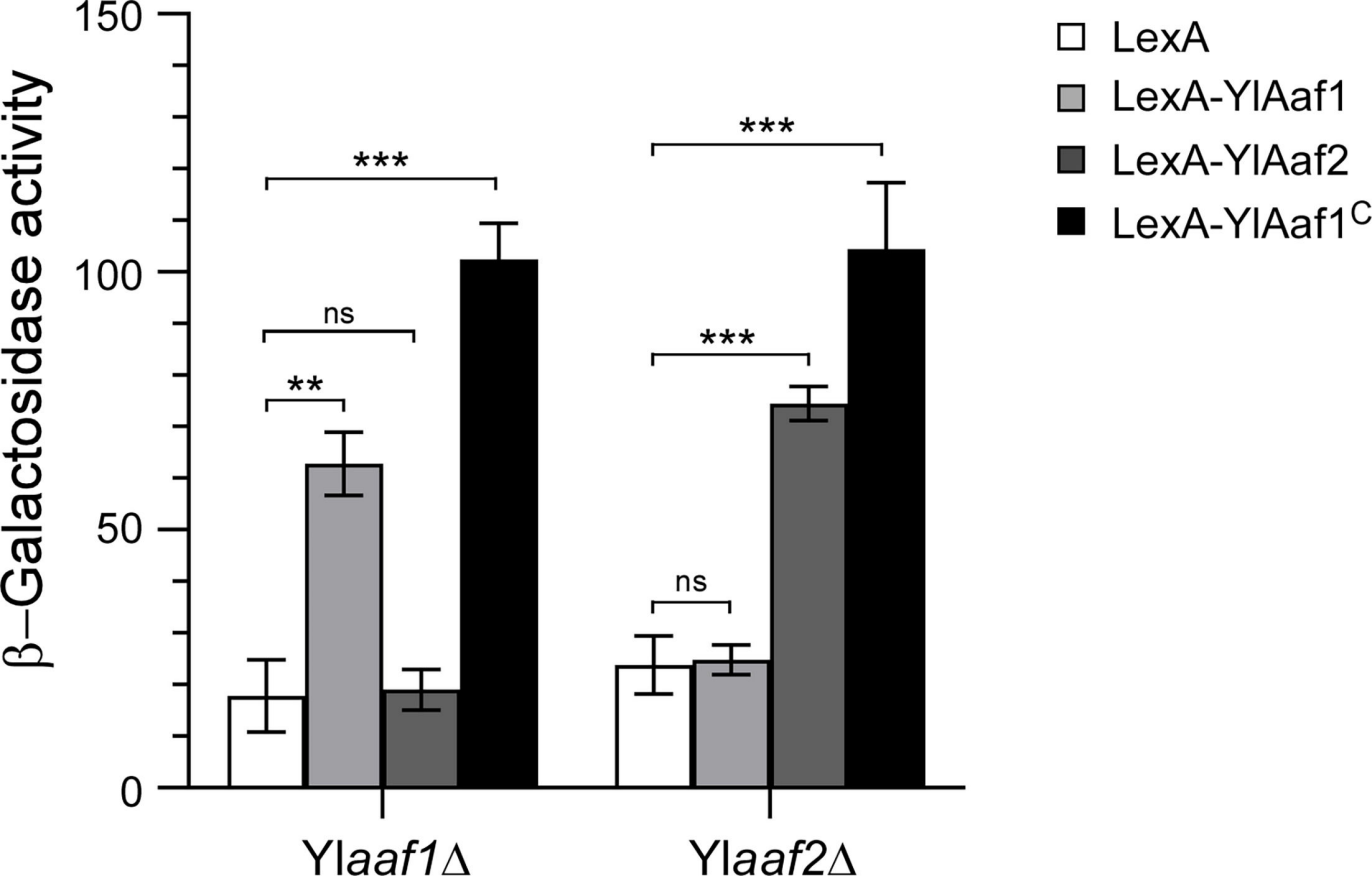
The YlAaf1-YlAaf2 complex has transcriptional activation activity. Plasmids pYL21-lexA, pYL21-lexA-YlAAF1, pYL21-lexA-YlAAF2, and pYL21-lexA-YlAAF1^244-355^ were transformed into Yl*aaf1*Δ and Yl*aaf2*Δ strains carrying reporter plasmid pINA445-lexAop-P_YlLEU2_-lacZ. The reporter gene contains the *lacZ* gene under the control of four copies of the *lexA* operator and Yl*LEU2* mini-promoter. Cells were grown in liquid YNBD (pH 7.5) medium. Cell lysates were measured for β-galactosidase activity.

To examine whether YlAaf1 and YlAaf2 may have transcriptional activation activity, the two proteins were fused to the DNA-binding domain of *E. coli* LexA (a.a. 1-87) and measured for their ability to influence *lexAop-P_YlLEU2_-lacZ* reporter gene expression in *Y. lipolytica*. When expressed in Yl*aaf1*Δ cells, LexA-YlAaf2 did not increase reporter expression. However, LexA-YlAaf1 increased reporter expression by 3.5-fold (Fig. 4). Interestingly, LexA-YlAaf1^C^ (containing the C-terminal region, a.a. 244-355) increased reporter expression by 5.7-fold (Fig. 4). When expressed in Yl*aaf2*Δ cells, LexA-YlAaf1 did not increase reporter expression. However, LexA-YlAaf2 increased reporter expression by 3.1-fold, and LexA-YlAaf1^C^ increased reporter expression by 4.4-fold (Fig. 4). This result suggests that the YlAaf1-YlAaf2 complex has transcription factor activity, which is mediated by the C-terminus of YlAaf1.

### YlAaf1-YlAaf2 is induced by alkaline pH and regulated by YlRim101 and Mhy1

**Fig 5:**
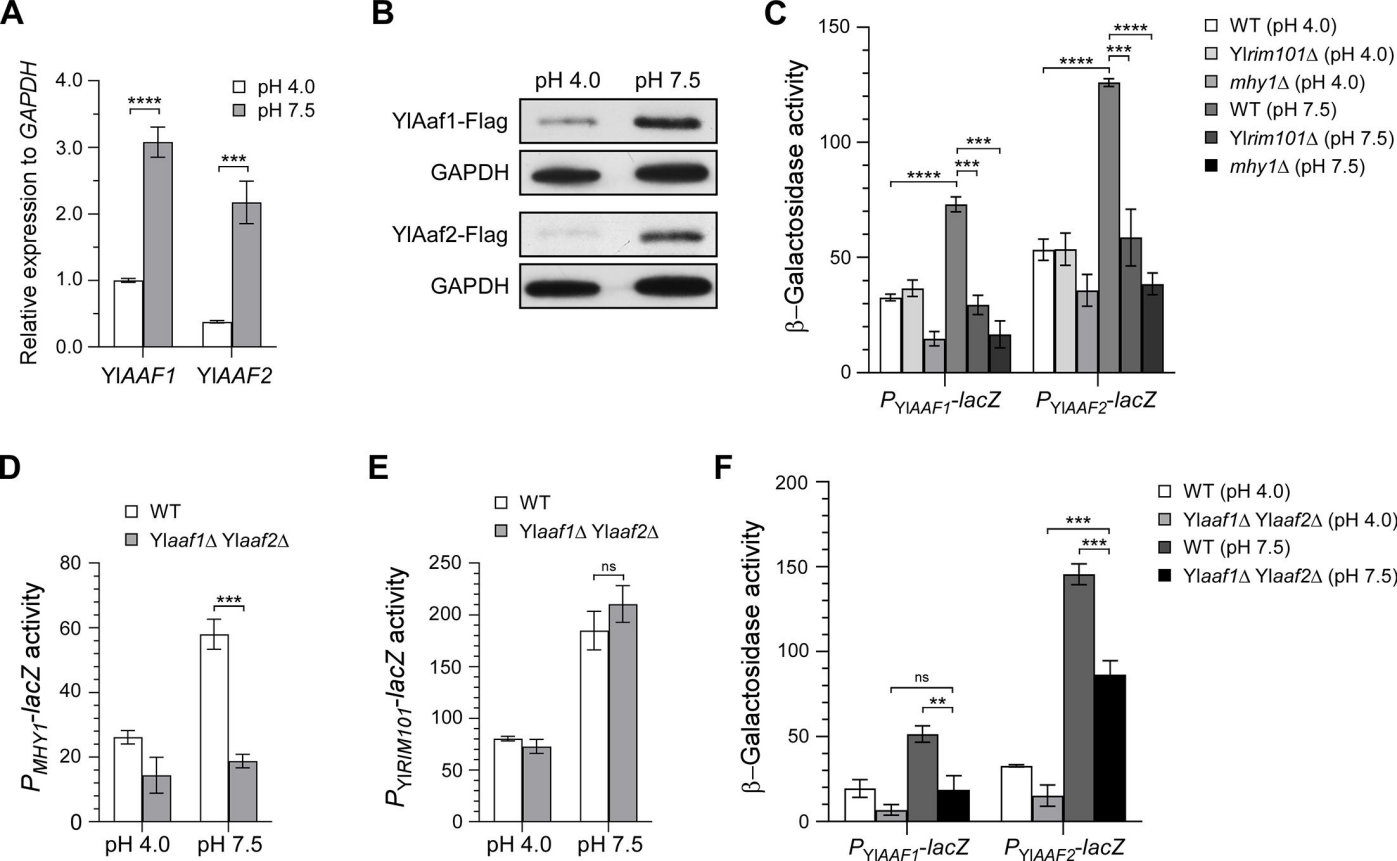
YlAaf1-YlAaf2 is induced by alkaline pH and regulated by YlRim101 and Mhy1. (**A**) The mRNA levels of Yl*AAF1* and Yl*AAF2* are higher at pH 7.5 than at pH 4.0. Cells of the wild-type strain carrying plasmid pINA445 were grown at 30°C for 16 h in liquid YNBG (pH 4.0) and YNBG (pH 7.5) media. The mRNA levels were determined by quantitative real-time PCR (qRT-PCR) and normalized to *GAPDH* mRNA. (**B**) The protein levels of YlAaf1-Flag and YlAaf2-Flag are higher at pH 7.5 than at pH 4.0. Cells of strain YLX554 (contains Yl*AAF1-3FLAG* at its chromosomal locus) and strain YLX555 (contains Yl*AAF2-3FLAG* at its chromosomal locus) carrying plasmid pINA445 were grown at 30°C for 16 h in liquid YNBG (pH 4.0) and YNBG (pH 7.5) media. The protein levels of YlAaf1-Flag and YlAaf2-Flag were determined by immunoblotting with an anti-Flag antibody. GAPDH protein was used as the control for normalization. (**C**) β-Galactosidase activities of *P*_Yl*AAF1*_-*lacZ* and *P*_Yl*AAF2*_-*lacZ* in cells of the wild-type, Yl*rim101*Δ, and *mhy1*Δ strains carrying pINA445-P_YlAAF1_-lacZ or pINA445-P_YlAAF2_-lacZ grown at 30°C for 16 h in liquid YNBG (pH 4.0) and YNBG (pH 7.5) media. (**D and E**) β-Galactosidase activities of *P_MHY1_-lacZ* (**D**) and *P*_Yl*RIM101*_-*lacZ* (**E**) in cells of the wild-type and Yl*aaf1*Δ Yl*aaf2*Δ strains carrying pINA445-P_MHY1_-lacZ or pINA445-P_YlRIM101_-lacZ grown at 30°C for 16 h in liquid YNBG (pH 4.0) and YNBG (pH 7.5) media. (**F**) β-Galactosidase activities of *P*_Yl*AAF1*_-*lacZ* and *P*_Yl*AAF2*_-*lacZ* in cells of the wild-type and Yl*aaf1*Δ Yl*aaf2*Δ strains carrying pINA445-P_YlAAF1_-lacZ or pINA445-P_YlAAF2_-lacZ grown at 30°C for 16 h in liquid YNBG (pH 4.0) and YNBG (pH 7.5) media. Statistically significant differences are indicated by the asterisks (**P < 0.01, ***P < 0.001, ****P < 0.0001), ns, not statistically significant.

Since YlAaf1-YlAaf2 is an activator of filamentation, we expect that the expression or activity of YlAaf1 and YlAaf2 might be upregulated during the yeast-to-filament transition. To test this idea, we measured the mRNA levels and protein levels of YlAaf1 and YlAaf2 at acidic pH (pH 4.0) and alkaline pH (pH 7.5), the latter of which is a potent inducer of filamentation in *Y. lipolytica*. Compared to pH 4.0, the mRNA levels of Yl*AAF1* and Yl*AAF2* increased 3.1-fold and 5.7-fold, respectively, at pH 7.5 (Fig. 5A). Similarly, the protein levels of YlAaf1 and YlAaf2 also increased substantially at pH 7.5 (Fig. 5B). This result indicates that both YlAaf1 and YlAaf2 are upregulated in expression under filament-inducing conditions. We also noted that the expression level of YlAaf2 was lower than that of YlAaf1 at pH 4.0 (Fig. 5A and B).

We reported previously that alkaline pH highly upregulated Yl*RIM101* and *MHY1*, two transcription factor genes that play critical roles in alkaline-induced filamentation (14). We found that both Yl*AAF1* and Yl*AAF2* exhibited a dramatic reduction in promoter activity in Yl*rim101*Δ and *mhy1*Δ cells at pH 7.5 (Fig. 5C), suggesting that the two Yl*AAF* genes are downstream targets of YlRim101 and Mhy1. Interestingly, the two YlAaf proteins also activate the expression of *MHY1* at pH 7.5 (Fig. 5D), but not that of Yl*RIM101* (Fig. 5E). Like YlRim101 and Mhy1, both of which activate their own expression (14), the two YlAaf proteins also activate their own expression as their transcription levels were markedly reduced in Yl*aaf1*Δ Yl*aaf2*Δ cells compared to those in wild-type cells at pH 7.5 (Fig. 5F).

Together, our results indicate that YlAaf1 and YlAaf2 are induced by alkaline pH, a potent inducer of filamentous growth. The two Yl*AAF* genes are also positively regulated by alkaline-induced transcription factors YlRim101, Mhy1, and themselves.

### Functional interactions between YlAaf1-YlAaf2, YlRim101, and Mhy1

**Fig 6:**
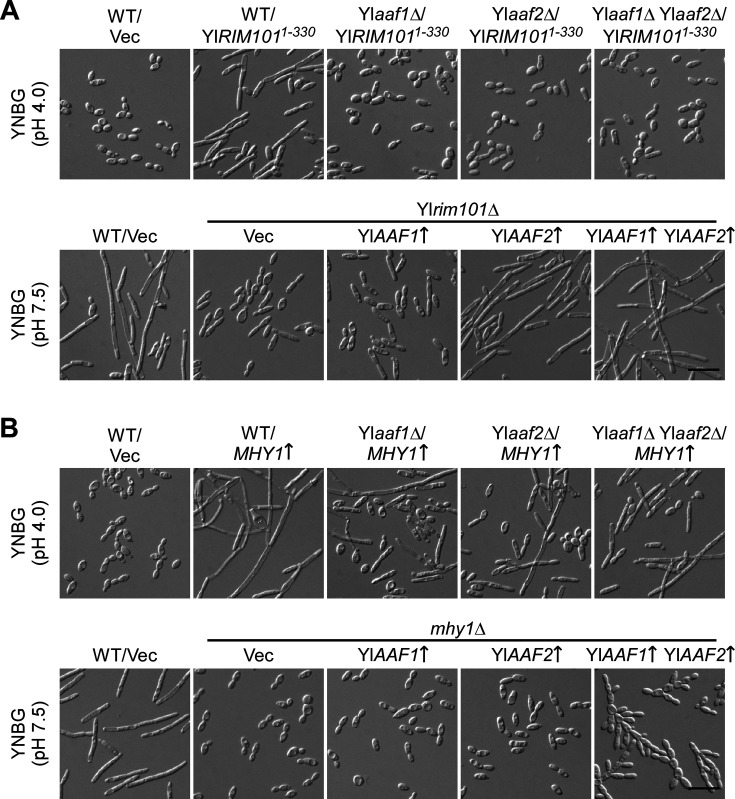
YlAaf1 and YlAaf2 are essential for YlRim101-regulated filamentation and partially required for Mhy1-regulated filamentation. (**A**) Cells of the wild-type strain carrying pINA445 (WT/Vec) and the wild-type, Yl*aaf1*Δ, Yl*aaf2*Δ, and Yl*aaf1*Δ Yl*aaf2*Δ strains carrying pINA445-YlRIM101^1-330^ were grown at 30°C for 16 h in liquid YNBG (pH 4.0) medium. Cells of the wild-type strain carrying pYL13 (WT/Vec) and Yl*rim101*Δ strain carrying pYL13 (Vec), pYL13-YlAAF1 (Yl*AAF1*↑), pYL13-YlAAF2 (Yl*AAF2*↑), or pYL13-YlAAF1-P_YlTEF1_-YlAAF2 (Yl*AAF1*↑ Yl*AAF2*↑) were grown at 30°C for 16 h in liquid YNBG (pH 7.5) medium. (**B**) Cells of the wild-type strain carrying pYL13 (WT/Vec) and the wild-type, Yl*aaf1*Δ, Yl*aaf2*Δ, and Yl*aaf1*Δ Yl*aaf2*Δ strains carrying pYL13-MHY1 (*MHY1*↑) were grown at 30°C for 16 h in liquid YNBG (pH 4.0) medium. Cells of the wild-type strain carrying pYL13 (WT/Vec) and the *mhy1*Δ strain carrying pYL13 (Vec), pYL13-YlAAF1 (Yl*AAF1*↑), pYL13-YlAAF2 (Yl*AAF2*↑), and pYL13-YlAAF1-P_YlTEF1_-YlAAF2 (Yl*AAF1*↑ Yl*AAF2*↑) were grown at 30°C for 16 h in liquid YNBG (pH 7.5) medium. Bars, 20 µm.

To examine the functional relationship of YlAaf1-YlAaf2 with YlRim101 and Mhy1, two key regulators of alkaline-induced filamentation, reciprocal epistatic analyses were performed. We reported previously that, at pH 4.0, where filamentation is not favored, the expression of YlRim101^1-330^ (a constitutively active mutant of YlRim101) stimulated strong filamentation in wild-type cells (14). We found that the expression of YlRim101^1-330^ failed to stimulate filamentation in Yl*aaf1*Δ, Yl*aaf2*Δ, and Yl*aaf1*Δ Yl*aaf2*Δ cells (Fig. 6A, top row). Forty percent of wild-type cells expressing YlRim101^1-330^ (*n* > 200) were longer than 20 µm, whereas less than 3% of wild-type cells carrying the empty vector, as well as Yl*aaf1*Δ, Yl*aaf2*Δ, and Yl*aaf1*Δ Yl*aaf2*Δ cells expressing YlRim101^1-330^ (*n* > 200) were longer than 20 µm. This result suggests that YlAaf1 and YlAaf2 are required for YlRim101-regulated filamentation. At pH 7.5, wild-type cells formed long filaments, whereas Yl*rim101*Δ cells were defective in filamentation. Eighty-two percent of wild-type cells carrying the empty vector (*n* > 200) were longer than 20 µm. In contrast, only 6% of Yl*rim101*Δ cells carrying the empty vector (*n* > 200) were longer than 20 µm (Fig. 6A, bottom row). However, the overexpression of Yl*AAF2* alone as well as the co-overexpression of Yl*AAF1* and Yl*AAF2* caused strong filamentation in Yl*rim101*Δ cells. Thirty-nine percent of Yl*rim101*Δ cells overexpressing Yl*AAF2* and 81% of Yl*rim101*Δ cells co-overexpressing Yl*AAF1* and Yl*AAF2* (*n* > 200) were longer than 20 µm. The overexpression of Yl*AAF1* alone did not significantly affect filamentation in Yl*rim101*Δ cells, as only 9% of Yl*rim101*Δ cells overexpressing Yl*AAF1* (*n* > 200) were longer than 20 µm. This result supports the idea that the two Yl*AAF* genes are targets of YlRim101.

The overexpression of *MHY1* strongly stimulated filamentation in wild-type cells at pH 4.0. Eighty-six percent of wild-type cells overexpressing *MHY1* (*n* > 200) were longer than 20 µm. In contrast, only 3% of wild-type cells carrying the empty vector (*n* > 200) were longer than 20 µm (Fig. 6B, top row). *MHY1* overexpression still caused the formation of filaments in Yl*aaf1*Δ, Yl*aaf2*Δ, and Yl*aaf1*Δ Yl*aaf2*Δ cells, albeit at reduced efficiency compared to wild-type cells (Fig. 6B, top row), as 41% of Yl*aaf1*Δ cells, 42% of Yl*aaf2*Δ cells, and 41% of Yl*aaf1*Δ Yl*aaf2*Δ cells overexpressing *MHY1* (*n* > 200) were longer than 20 µm. This finding suggests that the YlAaf proteins are partially required for Mhy1-regulated filamentation. As reported before (14), *mhy1*Δ cells were severely defective in filamentation and did not form any filaments at pH 7.5 (Fig. 6B, bottom row). Eighty-two percent of wild-type cells carrying the empty vector (*n* > 200) were longer than 20 µm. In contrast, none of the *mhy1*Δ cells carrying the empty vector (*n* > 200) were longer than 20 µm. The overexpression of Yl*AAF2* or Yl*AAF1* still did not stimulate filamentation in *mhy1*Δ cells, as none of the *mhy1*Δ cells overexpressing either Yl*AAF1* or Yl*AAF2* (*n* > 200) were longer than 20 µm. However, the co-overexpression of Yl*AAF1* and Yl*AAF2* caused the formation of pseudohyphae in *mhy1*Δ cells (Fig. 6B, bottom row). Fifty-seven percent of *mhy1*Δ cells co-overexpressing Yl*AAF1* and Yl*AAF2* (*n* > 200) were longer than 20 µm. This finding indicates that Mhy1 is largely required for YlAaf-stimulated filamentation, suggesting that Mhy1 is more potent in stimulating filamentation than YlAaf proteins.

Together, our results indicate that YlAaf1 and YlAaf2 are essential for YlRim101-regulated filamentation but are only partially required for Mhy1-regulated filamentation.

### YlAaf1-YlAaf2 and Mhy1 are direct regulatory targets of each other

**Fig 7:**
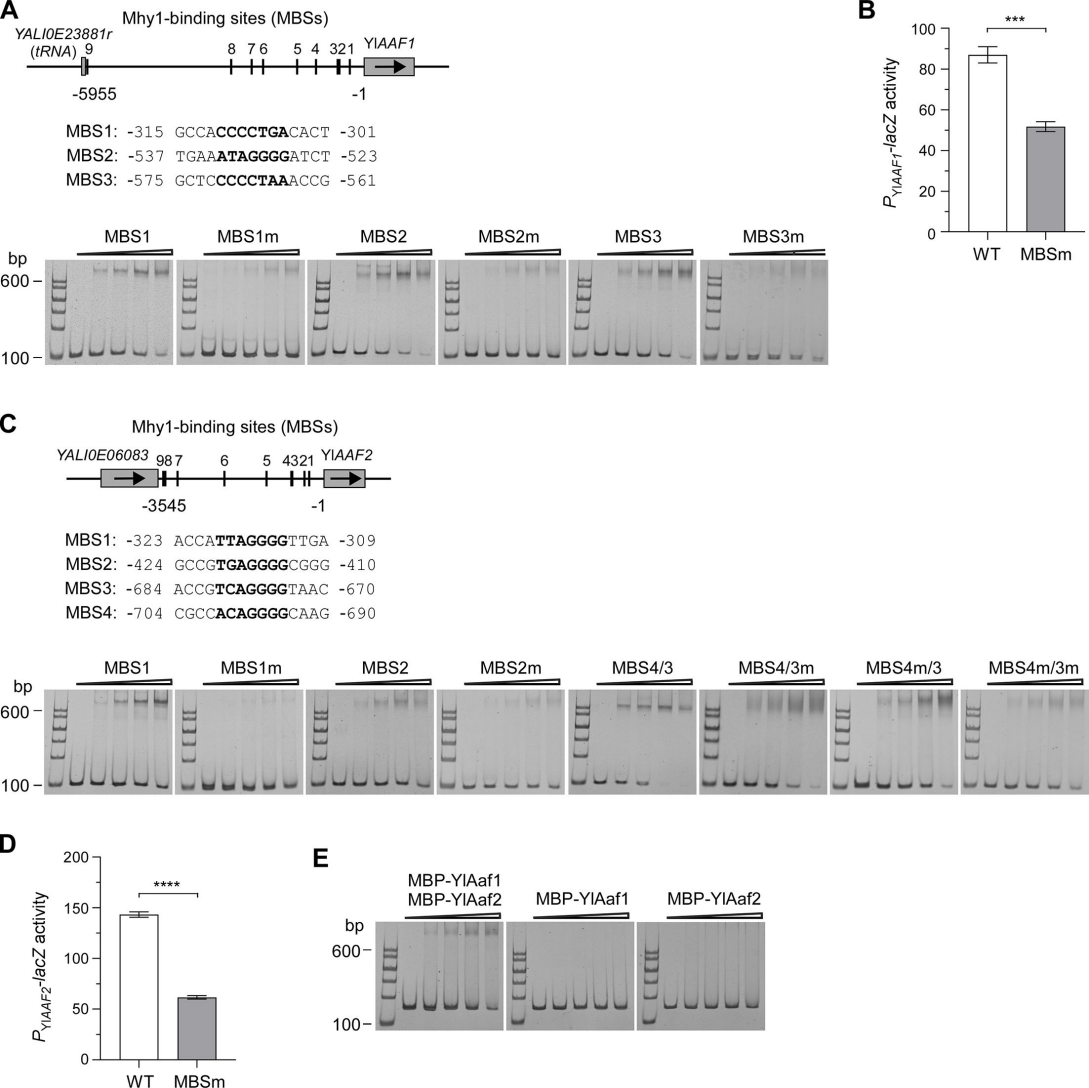
YlAaf1-YlAaf2 and Mhy1 are direct regulatory targets of each other. (**A**) Mhy1 binds to the MBS1, MBS2, and MBS3 motifs in the Yl*AAF1* promoter. Top panel, the position and nucleotide sequence of MBSs in Yl*AAF1* promoter. The gene that is located immediately upstream of Yl*AAF1* is a tRNA gene. Bottom panel, the binding of GST-Mhy1-C152 to MBS1, MBS2, and MBS3 and their mutants (MBSm) in EMSA. (**B**) Transcriptional activities of the 5,955-bp Yl*AAF1* promoter (WT) and its mutated form (MBSm) carrying MBS1, MBS2, and MBS3 mutations in wild-type strain grown at 30°C for 16 h in liquid YNBG (pH 7.5) medium. (**C**) Mhy1 binds to the MBS1, MBS2, MBS3, and MBS4 motifs in Yl*AAF2* promoter. Top panel, the position and nucleotide sequence of MBSs in Yl*AAF2* promoter. Bottom panel, the binding of GST-Mhy1-C152 to MBS1, MBS2, MBS3, and MBS4 and their mutants (MBSm) in EMSA. (**D**) Transcriptional activities of the 3,545-bp Yl*AAF2* promoter (WT) and its mutated form (MBSm) carrying MBS1, MBS2, MBS3, and MBS4 mutations in wild-type strain grown at 30°C for 16 h in liquid YNBG (pH 7.5) medium. Statistically significant differences are indicated by the asterisks (***P < 0.001, ****P < 0.0001). (**E**) The YlAaf1-YlAaf2 complex, but not YlAaf1 or YlAaf2 alone, binds to the 150-bp region (−775 to −626 bp) in the *MHY1* promoter.

We reported previously that Mhy1 binds to a specific STRE motif, 5′-WNAGGGG-3′ (Mhy1-binding site, MBS), in the promoter of target genes (30). To examine whether Yl*AAF1* and Yl*AAF2* are direct targets of Mhy1, an electrophoretic mobility shift assay (EMSA) was performed using bacterially expressed GST-Mhy1-C152 (a.a. 134-285) fusion protein, which has a stronger ability to bind MBS than full-length Mhy1 (30). There are nine potential MBSs in the 5,955-bp intergenic region upstream of the Yl*AAF1* ORF (Fig. 7A). We tested only the three MBSs that are located within the 1,000-bp sequence upstream of the Yl*AAF1* ORF. Mhy1 (refers to Mhy1-C152 for simplicity) bound to all DNA probes that contain either MBS1, MBS2, or MBS3, whereas single-nucleotide substitution (WNAGGGG→WNCGGGG) in MBS1, MBS2, or MBS3 dramatically reduced Mhy1 binding (Fig. 7A). To assess whether the three MBSs are important for Yl*AAF1* expression, single-nucleotide substitutions in all three MBSs were introduced into the 5,955-bp promoter of Yl*AAF1*, and its transcriptional activity was monitored using *P*_Yl*AAF1*_-*lacZ* reporter. We found that the nucleotide substitutions markedly reduced *P*_Yl*AAF1*_-*lacZ* expression in wild-type cells (Fig. 7B). These results suggest that Yl*AAF1* is a direct regulatory target of Mhy1.

Yl*AAF2* is separated from the upstream neighboring gene by a 3,545-bp intergenic region, which contains nine MBSs (Fig. 7C). Within the 1,000-bp region upstream of the Yl*AAF2* ORF, there are four MBSs. Among them, MBS3 and MBS4 are very close (separated by only 13 bp) and therefore included in one DNA probe. We found that Mhy1 bound to MBS1, MBS4/MBS3, and weakly to MBS2 (Fig. 7C). Single-nucleotide substitution (WNAGGGG→WNCGGGG) in MBS1 and MBS2 strongly reduced Mhy1 binding. Nucleotide substitution in both MBS3 and MBS4 of the MBS4/MBS3 motif also strongly reduced Mhy1 binding, whereas nucleotide substitution of either MBS3 or MBS4 alone did not drastically impair Mhy1 binding (Fig. 7C). This result suggests that Mhy1 binds to all four MBS sites. Single-nucleotide substitutions of all four MBSs markedly reduced *P*_Yl*AAF2*_-*lacZ* expression in wild-type cells (Fig. 7D). These results indicate that Yl*AAF2* is also a direct regulatory target of Mhy1.

To explore whether YlAaf1 and YlAaf2 may bind to the promoter of *MHY1*, MBP-tagged YlAaf1 and YlAaf2 were individually expressed in *E. coli* cells and purified for EMSA. We found that the YlAaf1-YlAaf2 complex bound to the 150-bp region (−775 to −626 bp) in the *MHY1* promoter. However, neither YlAaf1 nor YlAaf2 alone bound to this region (Fig. 7E). This result suggests that *MHY1* is a direct regulatory target of YlAaf1-YlAaf2, and YlAaf1 or YlAaf2 alone may not bind to DNA.

## DISCUSSION

The dimorphic yeast *Y. lipolytica* undergoes the yeast-to-filament transition when environmental conditions become unfavorable. This process requires a number of transcription factors that respond to environmental stimulations and activate downstream target genes important for this morphological transition. In this study, we identified YlAaf1 and YlAaf2, a pair of novel SANT domain-containing transcription factors that promote filamentous growth in *Y. lipolytica*. We showed that YlAaf1 and YlAaf2 are interdependent for function and form a complex that has transcriptional activation activity. We also showed that YlAaf1 and YlAaf2 respond to alkaline pH, a strong inducer of the yeast-to-filament transition, and are positively regulated by YlRim101 and Mhy1, two key regulators of alkaline-induced filamentation. Moreover, we showed that the YlAaf1-YlAaf2 complex and Mhy1 are direct regulatory targets of each other. Our results provide new insights into the regulatory network that governs the control of filamentation.

**Fig 8:**
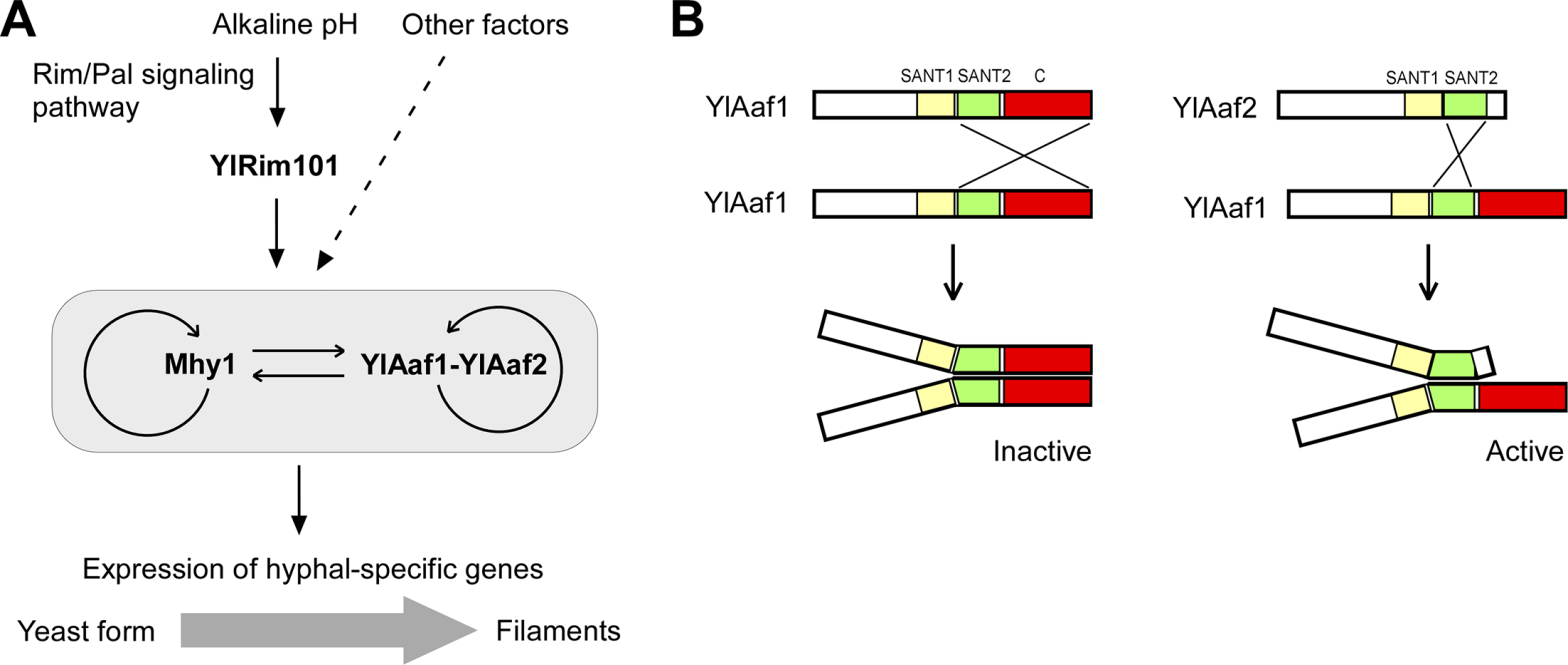
Models that explain the regulatory relationship between YlRim101, Mhy1, and YlAaf1-YlAaf2 and how the YlAaf1-YlAaf2 complex exhibits transcriptional activation activity. (**A**) A model that explains the regulatory relationship between YlRim101, Mhy1, and YlAaf1-YlAaf2. Both Mhy1 and YlAaf1-YlAaf2 autoregulate themselves transcriptionally. (**B**) A model that explains how the YlAaf1-YlAaf2 complex exhibits transcriptional activation activity. Left panel, YlAaf1 interacts with other YlAaf1 via SANT2 and the C-terminus. This interaction blocks its transcriptional activation activity. Right panel, YlAaf2 interacts with YlAaf1 via their respective SANT2 domains. This interaction frees the C-terminus of YlAaf1, releasing its transcriptional activation activity.

Previous studies suggest that Mhy1, a C_2_H_2_-type zinc finger transcription factor, is the master regulator of the yeast-to-filament transition in *Y. lipolytica*, as its deletion abolished filamentation under any of the filament-inducing conditions tested and its overexpression caused hyperfilamentation even under non-filament-inducing conditions (13, 30). In addition, the expression of *MHY1* highly responds to environmental stimulations as *MHY1* is one of the major targets of both positive regulation by alkaline pH and glucose as well as negative regulation by TORC1-Sch9 signaling and transcriptional repressors including Fts1, Fts2, and YlTup1-YlSsn6 (14, 18–20). Like *MHY1*, Yl*AAF1,* and Yl*AAF2* are also targets of positive regulation by alkaline pH and negative regulation by Fts1, Fts2, and YlTup1-YlSsn6 (14, 18, 19). Both *MHY1* and Yl*AAF1*-Yl*AAF2* genes are targets of YlRim101, the downstream effector of the Rim/Pal signaling pathway that senses alkaline pH (Fig. 8A). More importantly, our results showed that the YlAaf1-YlAaf2 complex and Mhy1 are direct targets of each other. These findings suggest that the YlAaf1-YlAaf2 complex might be another key regulatory hub that responds to various environmental stimulations (Fig. 8A). Like Mhy1, the YlAaf1-YlAaf2 complex may also promote filamentation through the activation of hyphal-specific genes. Since the co-overexpression of YlAaf1 and YlAaf2 stimulated filamentation to some extent (cells formed pseudohyphae) in the absence of Mhy1, the downstream target genes regulated by YlAaf1-YlAaf2 and Mhy1 might be partially overlapping.

YlAaf1 and YlAaf2 are closely related to *C. albicans* Aaf1, which also appears to affect filamentation (28). In *C. albicans*, the expression of the *AAF1* gene is induced by several growth conditions, including alkaline pH (31). In addition, Ca*AAF1* is the target of Efg1 under different conditions, including gut commensalism, biofilm formation, and white-opaque transition (32). CaAaf1 was thought to be a regulatory protein rather than an adhesin because CaAaf1 localized to the cytoplasm and nucleus but not to the cell wall in *S. cerevisiae* (26). In this paper, we show that GFP-tagged YlAaf1 and YlAaf2 also localize to the nucleus. In addition, we show that the C-terminus of YlAaf1 has transcriptional activation activity. Moreover, bacterially expressed YlAaf1-YlAaf2 complex binds directly to the promoter of *MHY1*. These results support the idea that Aaf1-related proteins are DNA-binding transcriptional regulators.

Although both Yl*AAF1* and Yl*AAF2* are upregulated by alkaline pH, there are some differences between these two Yl*AAF* genes. The expression level of Yl*AAF2* is much lower than that of Yl*AAF1* at acidic pH, a non-filament-inducing condition. At alkaline pH, the expression level of Yl*AAF2* increases more sharply than that of Yl*AAF1*, suggesting that Yl*AAF2* is more responsive to induction by alkaline pH. Human c-Myb has three DNA-binding domains. It can form a homodimer via a leucine zipper. However, homodimerization abolished its ability to bind DNA and is an important negative regulatory mechanism to control its activity (33). In light of this observation and our own results, we propose that under non-filament-inducing conditions, very few YlAaf2 proteins are produced in the cells compared to YlAaf1. An excess of YlAaf1 forms a homodimer, which is mediated by both the SANT2 domain and the C-terminus. This dimerization may somehow mask the transcriptional activation activity of the C-terminus (Fig. 8B, left panel). Under filament-inducing conditions, the expression levels of YlAaf1 and YlAaf2 increase rapidly. YlAaf2 binds to YlAaf1 via their SANT2 domains, and more YlAaf1-YlAaf2 complex is produced in the cells. The binding of YlAaf2 to YlAaf1 frees the C-terminus of YlAaf1 and releases its transcriptional activation activity (Fig. 8B, right panel).

Within the YlAaf1-YlAaf2 complex, YlAaf1 appears to play a major role since the C-terminus of YlAaf1 has transcriptional activation activity while the N-terminus (a.a. 1-118) of YlAaf1 is also important for filamentation (data not shown). In contrast, the functional region of YlAaf2 appears to be the two SANT domains as the N-terminus (a.a. 1-149) of YlAaf2 is dispensable for filamentation (data not shown). YlAaf2 may play a regulatory role in the activation of YlAaf1. Both the DNA-binding activity and transcriptional activation activity of YlAaf1 appear to be blocked by homotypic interaction between YlAaf1 and another YlAaf1. The binding of YlAaf2 to YlAaf1 may not only release the transcriptional activation activity of YlAaf1’s C-terminus, but also enable the binding to specific DNA sequences, probably by providing another SANT1 domain.

Aaf1-related proteins are widespread in yeasts, although some yeasts that lack them also exist, such as *S. cerevisiae*. Most yeast species, including *C. albicans,* have only one Aaf1-related protein. Interestingly, *Y. lipolytica* and a few other yeast species have two Aaf1-related proteins. YlAaf1 and YlAaf2 are unique in that they are not simply redundant in function but interdependent for function in the control of filamentation. Because the YlAaf1-YlAaf2 complex bound to a region in the promoter of *MHY1,* whereas neither YlAaf1 nor YlAaf2 alone bound to the same region, it raises a question of how the single Aaf1-related protein in other yeasts, including *C. albicans*, activates gene expression. In humans, the transcription factor c-Myc contains the DNA-binding domain. However, full-length c-Myc does not bind DNA. c-Myc was found to heterodimerize with Max, a protein that cooperates with c-Myc to bind specifically to a core DNA sequence (34). Similarly, the single Aaf1-related protein in other yeasts may heterodimerize with a yet unknown protein to activate gene expression.

In this study, we identified the YlAaf1-YlAaf2 transcriptional activator and characterized its role in regulating filamentous growth. However, many questions remain unanswered. For example, whether YlAaf1 and YlAaf2 may have other cellular functions? What environmental factors, apart from alkaline pH, may influence the expression of YlAaf1 and YlAaf2? How does the YlAaf1-YlAaf2 complex bind to DNA, and what DNA sequence does it recognize? Whether YlAaf2 may participate in DNA binding? We also did not determine whether YlAaf1 and YlAaf2 form a heterodimer or a heterooligomer. Future investigations are needed to answer these questions.

## MATERIALS AND METHODS

### Strains and media

*Y. lipolytica* strains used in this study are listed in Table S1 in the supplemental material. PO1a (*MATA leu2-270 ura3-302*) was used as the wild-type strain. Culture media include yeast extract-peptone-dextrose (YPD) medium (20 g/L peptone, 10 g/L yeast extract, 2% glucose), YNBD medium (6.7 g/L yeast nitrogen base without amino acid, 1% glucose), YNBG medium (6.7 g/L yeast nitrogen base without amino acid, 1% glycerol), YNBL medium (6.7 g/L yeast nitrogen base without amino acid, 1% sodium lactate), and yeast nitrogen base-dextrose-citrate-pH 7.0 medium (6.7 g/L yeast nitrogen base without amino acid, 294 mg/L trisodium citrate dehydrate, 1% glucose, pH 7.0) supplemented with 80 mg/L of leucine, 20 mg/L of uracil, or both when required. YNBG medium was buffered to pH 4.0 or 7.5 with Na_2_HPO_4_-citric acid buffer after autoclaving. For solid media, agar was added to 2%. Serum agar (1% newborn calf serum, 2% agar). *S. cerevisiae* strains used in this study are listed in Table S2. Standard culture media were used for *S. cerevisiae* (35). The *E. coli* strains DH5α and BL21(DE3) were used for plasmid amplification and recombinant protein production, respectively.

### Plasmid construction

The plasmids and oligonucleotides used in this study are listed in Tables S3 and S4, respectively. To generate pINA445-P_5955 bp_-YlAAF1, two steps were employed. First, Yl*AAF1* carrying a 376-bp 3′-UTR was amplified by PCR and inserted into *Hin*dIII-digested vector pINA445 (*CEN*, *LEU2*) using ClonExpress II One Step Cloning Kit (Vazyme Biotech Co., China). Then, the 5,955-bp promoter of Yl*AAF1* was amplified by PCR and inserted into the *Cla*I-digested previous vector. Similarly, Yl*AAF2* carrying a 441-bp 3′-UTR and the 3,545-bp promoter of Yl*AAF2* was inserted into pINA445, yielding pINA445-P_3545 bp_-YlAAF2. pINA445-YlRIM101^1-330^ was reported previously (14). To overexpress Yl*AAF1* and Yl*AAF2*, the ORF of each gene plus the 500-bp 3′-UTR was amplified by PCR and inserted into *Hin*dIII-digested vector pYL13 (*CEN*, *LEU2*, *P_YlTEF1_*) (17), yielding pYL13-Gene. pYL13-MHY1 was reported previously (30). To co-overexpress Yl*AAF1* and Yl*AAF2*, Yl*AAF1* plus the 500-bp 3′-UTR was amplified by PCR and inserted into *Sma*I-digested vector pYL13. The resulting plasmid was digested by *Hin*dIII, and *P*_Yl*TEF1*_-Yl*AAF2* amplified from pYL13-YlAAF2 was inserted into this plasmid, with Yl*AAF1* and Yl*AAF2* facing each other.

To examine the localization of YlAaf1 and YlAaf2 in *Y. lipolytica* cells, Yl*AAF1* carrying the 5,955-bp promoter and Yl*AAF2* carrying the 3,545-bp promoter were amplified by PCR and inserted into *Bam*HI-digested vector pYL14 (pINA445 backbone, Yl*LEU2*, *GFP-T_YlURA3_*) (17), yielding pYL14-YlAAF1 and pYL14-YlAAF2 that express YlAaf1-GFP and YlAaf2-GFP, respectively.

For the BiFC assay in *S. cerevisiae*, the ORF of Yl*AAF1* was amplified by PCR and inserted into the *Eco*RI-digested vector pVN1 (*CEN*, *URA3*, *P_MET25_-Venus-N-T_CYC1_*) (36), yielding pVN1-YlAAF1. Similarly, the ORF of Yl*AAF2* was amplified by PCR and inserted into *Eco*RI-digested vector pVC1 (*CEN*, *HIS3*, *P_MET25_-Venus-C-T_CYC1_*) (36), yielding pVC1-YlAAF2.

For yeast two-hybrid assay, Yl*AAF1* segments were inserted into *Bam*HI-digested vectors pGAD-C1 (2μ, *LEU2*, *GAL4-AD*) and pGBDU-C1 (2μ, *URA3*, *GAL4-DBD*) (37), yielding pGAD-YlAAF1 segments and pGBDU-YlAAF1 segments, respectively. Similarly, Yl*AAF2* segments were inserted into *Bam*HI-digested vector pGAD-C1 and pGBDU-C1, yielding pGAD-YlAAF2 segments and pGBDU-YlAAF2 segments, respectively.

For the GST pull-down assay, Yl*AAF1* and Yl*AAF2* segments were fused to *GST* by overlapping PCR, then inserted into *Eco*RI-digested vector pMAL-c2X, yielding pMAL-YlAAF1 segments-GST and pMAL-YlAAF2 segments-GST, respectively. *GST* was inserted into the *Eco*RI-digested vector pMAL-c2X, yielding pMAL-GST as a negative control. To generate pMAL-YlAAF1-FLAG and pMAL-YlAAF2-FLAG, Yl*AAF1* and Yl*AAF2* were fused to *FLAG* by overlapping PCR, then inserted into the *Eco*RI-digested vector pMAL-c2X, respectively.

For one-hybrid assay in *Y. lipolytica*, the DNA-binding domain (a.a. 1-87) of the *E. coli lexA* gene was amplified by PCR and inserted into *Hin*dIII-digested vector pYL21 (*CEN*, Yl*URA3*, *P*_Yl*TEF1*_) (14), yielding pYL21-lexA. Similarly, Yl*AAF1*, Yl*AAF1*^244-355^, and Yl*AAF2* were fused to *lexA* by overlapping PCR and inserted into *Hin*dIII-digested vector pYL21, yielding pYL21-lexA-YlAAF1, pYL21-lexA-YlAAF1^244-355^, and pYL21-lexA-YlAAF2. The reporter plasmid pINA445-lexAop-P_YlLEU2_-lacZ was reported previously (17).

To generate pINA445-P_YlAAF1_-lacZ and pINA445-P_YlAAF2_-lacZ, the 5,955-bp Yl*AAF1* promoter and 3,545-bp Yl*AAF2* promoter were amplified by PCR and inserted into *Hin*dIII-digested vector pINA445-lacZ (17), respectively. pINA445-P_YlRIM101_-lacZ and pINA445-P_MHY1_-lacZ were reported previously (14, 30). Mutations of Mhy1-binding sites (MBSs) in the promoter region of Yl*AAF1* and Yl*AAF2* were done by overlapping PCR.

To generate MBP-YlAaf1 and MBP-YlAaf2 for EMSA, the ORF of Yl*AAF1* and Yl*AAF2* was amplified by PCR and inserted into the *Eco*RI-digested vector pMAL-c2X individually. The plasmid used for producing GST-Mhy1-152 aa was reported previously (30).

### Yeast strain construction

Yl*AAF1* and Yl*AAF2* were deleted in *Y. lipolytica* strains by homologous recombination. Briefly, the ~1.0-kb sequence upstream of the gene ORF (P_gene_) and the ~1.0-kb sequence downstream of the gene ORF (T_gene_) were amplified by PCR from genomic DNA and then fused with *loxR*-Yl*URA3-loxP* and Yl*LEU2* that were amplified by overlapping PCR from pYL8 (17). The resulting *P_gene_-loxR-*Yl*URA3-loxP-T_gene_-*Yl*LEU2* deletion cassette was used to transform yeast cells. Yeast transformants were examined by PCR to identify the ones bearing the correct gene deletion. The Yl*URA3* marker was later removed by Cre-mediated DNA recombination between *loxR* and *loxP* sites. To tag the chromosomal copy of Yl*AAF1* with a 3*FLAG* tag, Yl*AAF1-3FLAG* was generated by overlapping PCR. Then, Yl*AAF1-3FLAG-loxR-*Yl*URA3-loxP-T*_Yl*AAF1*_*-*Yl*LEU2* was constructed and used to tag the chromosomal copy of Yl*AAF1* C-terminally with three copies of *FLAG* by homologous recombination, yielding the yeast strain YLX554 (Yl*AAF1-3FLAG:loxR/P*). Strain YLX555 (Yl*AAF2-3FLAG:loxR/P*) was constructed similarly.

### Yeast two-hybrid assay

pGAD-C1-based plasmids were transformed into the *S. cerevisiae* strain pJ69-4A. pGBDU-C1-based plasmids were transformed into the *S. cerevisiae* strain pJ69-4α. Pairs of strains were mated on YPD plates and then replica plated onto SC-Leu-Ura plates to select for diploid cells that harbor both bait and prey plasmids. Diploid cells were patched on SC-Leu-Ura plates and replica plated onto SC-Leu-Ura-His plates to check for growth. Growth indicates interaction between the DBD and AD fusion proteins.

### Production of MBP-YlAaf-GST and MBP-YlAaf-Flag fusion proteins in *E. coli*

Each fusion protein contains two tags, MBP/GST or MBP/Flag. The MBP tag was added to increase protein solubility. Cells of *E. coli* strain BL21 (DE3) carrying expression plasmids were inoculated into liquid LB with ampicillin and grown overnight at 37°C. The culture was diluted 1:100 into fresh LB-ampicillin and grown at 37°C until OD_600_ reached 0.6. Isopropyl-β-D-thiogalactoside was added at 1 mM concentration, and the cells were grown for 12 h at 16°C. Cells were collected, washed, suspended in wash buffer (50 mM Tris-HCl, 0.5 mM NaCl, 1 mM DTT, pH 7.5), and lysed by ultrasonication.

### GST pull-down assay

* E. coli* cell lysates containing GST-tagged proteins were added to gravity columns containing Glutathione-Sefinose resin (BBI Life Science, China). Resins were washed with wash buffer. Then, *E. coli* cell lysates containing Flag-tagged proteins were added to the gravity column and incubated overnight at 4°C. Resins were washed, and elution buffer (1.538 mg/mL GSH, pH 7.9–8.1) was used to elute bound proteins. GST-tagged proteins were examined by SDS-PAGE followed by staining with Coomassie blue. Flag-tagged proteins were detected by immunoblotting.

### RNA extraction and quantitative real-time PCR analysis

Yeast cells grown at 30°C were collected at an OD_600_ of ~0.8. Total RNAs were extracted using Yeast RNA Kit (Omega, China). RNA integrity was examined by 1% agarose gel electrophoresis. Using a NanoDrop One Spectrometer (Thermo Scientific, USA), the yields of RNAs were examined, and the A260/A280 ratios that reflect the quality of RNAs were determined. A 1 µg total RNA per sample was used for reverse transcription using the HiScript II Q RT SuperMix for quantitative PCR (qPCR) (Vazyme Biotech Co., China). qPCRs were carried out using ChamQ Universal SYBR qPCR Master (Vazyme Biotech Co., China) following the manufacturer’s instructions. Triplicates were analyzed for each sample using Bio-Rad CFX Maestro (version 2.2) with normalized mode (ΔΔCq).

### Detection of YlAaf1-Flag and YlAaf2-Flag in *Y. lipolytica* cells

Cells of *Y. lipolytica* strains carrying Yl*AAF1-3FLAG* or Yl*AAF2-3FLAG* at its chromosomal locus and plasmid pINA445 were grown in liquid YNBG (pH 4.0) and YNBG (pH 7.5) media. Soluble proteins were extracted by the NaOH/TCA method (38). Standard immunoblotting procedures were used. Primary antibodies used were mouse monoclonal antibodies against Flag and against GAPDH (Proteintech, Rosemont, IL, USA). Horseradish peroxidase-conjugated goat anti-mouse IgG (Biofly Corporation, China) was used as the secondary antibody.

### β-Galactosidase assay

The β-galactosidase activity in the cells was determined by the crude cell extract assay with o-nitrophenyl-β-D-galactopyranoside (ONPG) as the substrate, as reported previously (17). Crude cell extracts were prepared by vortexing with glass beads. Protein concentration in the cell extracts was measured by the Bradford method. The specific β-galactosidase activity was normalized by the amount of total protein in each extract and was calculated according to the following formula: U = (OD_420_ × 1.7)/(0.0045 × protein concentration [mg mL^−1^] × sample vol [mL] × time [min]). The assays were performed in triplicate.

### Electrophoretic mobility shift assay

Glutathione-Sefinose resin was used to purify GST-Mhy1-C152, whereas amylose resin (BBI Life Science, China) was used to purify MBP-YlAaf1 and MBP-YlAaf2. The concentration of proteins in the eluate was quantified by the Bradford method and was adjusted to 0.1 mg/mL. Purified proteins were also examined by SDS-PAGE followed by staining with Coomassie blue. DNA probes for EMSA were amplified by PCR and purified by using the Gel Extraction Kit (Omega, China). To mutate Mhy1-binding sites (MBSs), the nucleotide A in WNAGGGG was mutated to C by overlapping PCR. The concentrations of DNA probes were measured by the NanoDrop One Spectrometer. For DNA–protein binding, DNA probe (300 ng) and recombinant protein (0, 0.0625, 0.125, 0.25, and 0.5 µg) were mixed in 20 µL binding buffer (10 mM Tris-HCl, 50 mM NaCl, 0.5 mM EDTA, 1 mM MgCl_2_, 4% glycerol, 0.5 mM dithiothreitol [DTT], pH 7.5) (39). DNA probe and DNA–protein complex were fractionated in 4% non-denaturing polyacrylamide gel in 0.5× Tris-Borate-EDTA buffer at 4°C. The gels were stained for DNA with ethidium bromide (EB).

### Microscopy

An Olympus BX51 microscope (Tokyo, Japan) and a Retiga 2000R CCD camera (QImaging Corporation, Canada) were used to visualize cell morphology and GFP. The images were acquired using QCapture Suite (QImaging Corporation, Canada). For the visualization of the nucleus, yeast cells were stained with 4′,6′-diamidino-2-phenylindole (DAPI) (Sigma-Aldrich, St Louis, MO) at 1 µg/mL.

## SUPPLEMENTAL MATERIAL

Table S1 to S4 msphere.00403-25-s0001.pdf Yeast strains, plasmids, and oligonucleotides used in this study.

ASM does not own the copyrights to Supplemental Material that may be linked to, or accessed through, an article. The authors have granted ASM a non-exclusive, world-wide license to publish the Supplemental Material files. Please contact the corresponding author directly for reuse.
