# Supplementary material for: YlAaf1-YlAaf2, a bipartite SANT domain-containing complex of transcriptional activator, promotes filamentous growth in the dimorphic yeast Yarrowia lipolytica
Source: mSphere. 2025 Aug 18;10(9):e00403-25. doi: 10.1128/msphere.00403-25 (PMC12482196; doi:10.1128/msphere.00403-25)
Supplement: Table S1 to S4 — Yeast strains, plasmids, and oligonucleotides used in this study. [file msphere.00403-25-s0001.pdf]

Supplementary material

Table S1. *Y. lipolytica* strains used in this study.

Table S2. *S. cerevisiae* strains used in this study.

Table S3. Plasmids used in this study.

Table S4. Oligonucleotides used in this study.

Table S1. ***Y. lipolytica*** **strains used in this study.**

Strain	Genotype	Source
PO1a	*MATA leu2-270 ura3-302*	1
YLX497	As PO1a except *mhy1Δ::loxR/P*	2
YLX514	As PO1a except Yl*rim101Δ::loxR/P*	3
YLX551	As PO1a except Yl*aaf1Δ::loxR/P*	This study
YLX552	As PO1a except Yl*aaf2Δ::loxR/P*	This study
YLX553	As PO1a except Yl*aaf1Δ::loxR/P* Yl*aaf2Δ::loxR/P*	This study
YLX554	As PO1a except Yl*AAF1-3FLAG:loxR/P*	This study
YLX555	As PO1a except Yl*AAF2-3FLAG:loxR/P*	This study

References

1. Barth G and Gaillardin C (1996) The dimorphic fungus *Yarrowia lipolytica*. p.313-368. In K. Wolf (ed). Non-conventional yeasts in biotechnology. Springer, Heidelberg, Germany.

2. Wu H, Shu T, Mao Y-S and Gao X-D (2020) Characterization of the promoter, downstream target genes and recognition DNA sequenced of Mhy1, a key filamentation-promoting transcription factor in the dimorphic yeast *Yarrowia lipolytica*. *Curr. Genet*. 66: 245-261.

3. Shu T, He X-Y, Chen J-W, Mao Y-S and Gao X-D (2021) The pH-responsive transcription factors YlRim101 and Mhy1 regulate alkaline pH-induced filamentation in the dimorphic yeast *Yarrowia lipolytica*. *mSphere*. 6: e00179-21.

Table S2. ***S. cerevisiae*** **strains used in this study.**

Strain	Genotype	Source
YEF473A	*MATa leu2-Δ1 his3-Δ200 lys2-801 trp1-Δ63 ura3-52*	1
pJ69-4A	*MATa his3-Δ200 leu2-3,112 trp1-901 ura3-52 gal4Δ gal80Δ LYS2::GAL1-HIS3 GAL2-ADE2 met2::GAL7-lacZ*	2
pJ69-4α	*MATα his3-Δ200 leu2-3,112 trp1-901 ura3-52 gal4Δ gal80Δ LYS2::GAL1-HIS3 GAL2-ADE2 met2::GAL7-lacZ*	2

References

1. Bi E and Pringle JR (1996) *ZDS1* and *ZDS2*, genes whose products may regulate Cdc42p in *Saccharomyces cerevisiae*. *Mol. Cell. Biol.* 16: 5264-5275.

2. Gao X-D, Albert S, Tcheperegine SE, Burd CG, Gallwitz D and Bi E (2003) The GAP activity of Msb3p and Msb4p for the Rab GTPase Sec4p is required for efficient exocytosis and actin organization. *J. Cell Biol.* 162: 635-646.

Table S3. Plasmids used in this study.

Plasmid	Description	Source
pYL8	pBlueScript KS(+) carrying *loxR*-Yl*URA3-loxP*	1
pRRQ2	*ARS68 (CEN/ARS)*, Yl*LEU2*, *hp4d-CRE*	2
pINA445	pBR322 carrying *ARS68 (CEN/ARS)* and Yl*LEU2*	3
pINA445-$P_{5955\ bp}$-YlAAF1	Yl*AAF1* carrying 5955-bp promoter and 376-bp 3'-UTR	This study
pINA445-$P_{3545\ bp}$-YlAAF2	Yl*AAF2* carrying 3545-bp promoter and 441-bp 3'-UTR	This study
pYL13	pINA445 carrying 406-bp Yl*TEF1* promoter	4
pYL13-YlAAF1	P_{YlTEF1}-Yl*AAF1* plus 500-bp 3'-UTR	This study
pYL13-YlAAF2	P_{YlTEF1}-Yl*AAF2* plus 500-bp 3'-UTR	This study
pYL13-YlAAF1-P_{YlTEF1}-YlAAF2	P_{YlTEF1}-Yl*AAF1* plus 500-bp 3'-UTR<br>P_{YlTEF1}-Yl*AAF2* plus 500-bp 3'-UTR	This study
pYL14	pINA445 carrying *EGFP*-T_{YlURA3}	1
pYL14-YlAAF1	Yl*AAF1-GFP* carrying 5955-bp Yl*AAF1* promoter	This study
pYL14-YlAAF2	Yl*AAF2-GFP* carrying 3545-bp Yl*AAF2* promoter	This study
pVN1	*CEN, URA3*, P_{MET25}-*Venus-N*-T_{CYC1}	5
pVN1-YlAAF1	P_{MET25}-Yl*AAF1-Venus-N*-T_{CYC1}	This study
pVC1	*CEN, HIS3*, P_{MET25}-*Venus-C*-T_{CYC1}	5
pVC1-YlAAF2	P_{MET25}-Yl*AAF2-Venus-C*-T_{CYC1}	This study
pGAD-C1	2μ, *LEU2*, *GAL4-AD*	6
pGAD-YlAAF1	*GAL4-AD*-Yl*AAF1*	This study
pGAD-YlAAF1$^{126\text{-}185}$	*GAL4-AD*-Yl*AAF1*$^{126\text{-}185}$	This study
pGAD-YlAAF1$^{178\text{-}243}$	*GAL4-AD*-Yl*AAF1*$^{178\text{-}243}$	This study
pGAD-YlAAF2	*GAL4-AD*-Yl*AAF2*	This study
pGAD-YlAAF2$^{157\text{-}216}$	*GAL4-AD*-Yl*AAF2*$^{157\text{-}216}$	This study
pGAD-YlAAF2$^{207\text{-}277}$	*GAL4-AD*-Yl*AAF2*$^{207\text{-}277}$	This study
pGBDU-C1	2μ, *URA3*, *GAL4-DBD*	6
pGBDU-YlAAF1	*GAL4-DBD*-Yl*AAF1*	This study
pGBDU-YlAAF1$^{126\text{-}185}$	*GAL4-DBD*-Yl*AAF1*$^{126\text{-}185}$	This study
pGBDU-YlAAF1$^{178\text{-}243}$	*GAL4-DBD*-Yl*AAF1*$^{178\text{-}243}$	This study
pGBDU-YlAAF2	*GAL4-DBD*-Yl*AAF2*	This study
pGBDU-YlAAF2$^{157\text{-}216}$	*GAL4-DBD*-Yl*AAF2*$^{157\text{-}216}$	This study
pGBDU-YlAAF2$^{207\text{-}277}$	*GAL4-DBD*-Yl*AAF2*$^{207\text{-}277}$	This study
pGEX-4T-1		
pMAL-c2X		
pMAL-GST	*GST* in pMAL-c2X	This study
pMAL-YlAAF1-GST	Yl*AAF1* plus *GST* in pMAL-c2X	This study
pMAL-YlAAF1$^{184\text{-}237\Delta}$-GST	Yl*AAF1*$^{184\text{-}237\Delta}$ plus *GST* in pMAL-c2X	This study
pMAL-YlAAF1$^{178\text{-}243}$-GST	Yl*AAF1*$^{178\text{-}243}$ plus *GST* in pMAL-c2X	This study
pMAL-YlAAF1$^{1\text{-}125}$-GST	Yl*AAF1*$^{1\text{-}125}$ plus *GST* in pMAL-c2X	This study
pMAL-YlAAF1$^{126\text{-}185}$-GST	Yl*AAF1*$^{126\text{-}185}$ plus *GST* in pMAL-c2X	This study
pMAL-YlAAF1$^{244\text{-}355}$-GST	Yl*AAF1*$^{244\text{-}355}$ plus *GST* in pMAL-c2X	This study

pMAL-YlAAF1$^{1-185}$-GST	Yl*AAF1*$^{1-185}$ plus *GST* in pMAL-c2X	This study
pMAL-YlAAF1$^{178-355}$-GST	Yl*AAF1*$^{178-355}$ plus *GST* in pMAL-c2X	This study
pMAL-YlAAF2-GST	Yl*AAF2* plus *GST* in pMAL-c2X	This study
pMAL-YlAAF2$^{213-267\Delta}$-GST	Yl*AAF2*$^{213-267\Delta}$ plus *GST* in pMAL-c2X	This study
pMAL-YlAAF2$^{207-277}$-GST	Yl*AAF2*$^{207-277}$ plus *GST* in pMAL-c2X	This study
pMAL-YlAAF1-FLAG	Yl*AAF1* plus *FLAG* in pMAL-c2X	This study
pMAL-YlAAF2-FLAG	Yl*AAF2* plus *FLAG* in pMAL-c2X	This study
pINA445-lexAop-P$_{YlLEU2}$-lacZ	*lexAop4-*P_{YlLEU2}*-lacZ* in pINA445	1
pYL21	*ARS68 (CEN/ARS),* Yl*URA3*, P_{YlTEF1}	7
pYL21-lexA	P_{YlTEF1}*-lexA*	This study
pYL21-lexA-YlAAF1	P_{YlTEF1}*-lexA*-Yl*AAF1*	This study
pYL21-lexA-YlAAF2	P_{YlTEF1}*-lexA*-Yl*AAF2*	This study
pYL21-lexA-YlAAF1$^{244-355}$	P_{YlTEF1}*-lexA*-Yl*AAF1*$^{244-355}$	This study
pINA445-lacZ	3.3 kb *lacZ* gene from plasmid pSH18-34	1
pINA445-P$_{YlAAF1}$-lacZ	5955-bp Yl*AAF1* promoter fused to *lacZ*	This study
pINA445-P$_{YlAAF2}$-lacZ	3545-bp Yl*AAF2* promoter fused to *lacZ*	This study
pINA445-P$_{YlRIM101}$-lacZ	5460-bp Yl*RIM101* promoter fused to *lacZ*	7
pINA445-P$_{MHY1}$-lacZ	4308-bp *MHY1* promoter fused to *lacZ*	8
pINA445-YlRIM101$^{1-330}$	Yl*RIM101*$^{1-330}$ encoding a.a. 1-330 with 2946-bp promoter and 282-bp 3'-UTR	7
pYL13-MHY1	P_{YlTEF1}*-MHY1* plus 327-bp 3'-UTR	8
GST-Mhy1-152 aa	*MHY1* ORF (+403, +858) in pGEX-4T-1	8
pMAL-YlAAF1	Yl*AAF1* ORF in pMAL-c2X	This study
pMAL-YlAAF2	Yl*AAF2* ORF in pMAL-c2X	This study
pINA445-P$_{YlAAF1}$m-lacZ	5955-bp Yl*AAF1* promoter carrying STRE1-STRE3 mutations	This study
pINA445-P$_{YlAAF2}$m-lacZ	3545-bp Yl*AAF2* promoter carrying STRE1-STRE4 mutations	This study

References

1. Zhao X-F, Li M, Li Y-Q, Chen X-D and Gao X-D (2013) The TEA/ATTS transcription factor YlTec1p represses the yeast-to-hypha transition in the dimorphic yeast *Yarrowia lipolytica*. *FEMS Yeast Res*. 13: 50-61.

2. Richard M, Quijano RR, Bezzate S, Bordon-Pallier F and Gaillardin C (2001) Tagging morphogenetic genes by insertional mutagenesis in the yeast *Yarrowia lipolytica*. *J. Bacteriol*. 183: 3098-3107.

3. Nuttley WM, Brade AM, Gaillardin C, Eitzen GA, Glover JR, Aitchison JD and Rachubinski RA (1993) Rapid identification and characterization of peroxisomal assembly mutants in *Yarrowia lipolytica*. *Yeast*. 9: 507–517.

4. Zhao X-F, Li M, Li Y-Q, Chen X-D and Gao X-D (2013) The TEA/ATTS transcription factor YlTec1p represses the yeast-to-hypha transition in the dimorphic yeast *Yarrowia lipolytica*. *FEMS Yeast Res*. 13: 50-61.

5. Gong T, Liao Y, He F, Yang Y, Yang D-D, Chen X-D and Gao X-D (2013) Control of polarized growth by

the Rho family GTPase Rho4 in budding yeast: requirement of the N-terminal extension of Rho4 and regulation by the Rho GTPase-activating protein Bem2. *Eukaryot Cell*. 12: 368-77.

6. James P, Halladay J and Craig EA (1996) Genomic libraries and a host strain designed for highly efficient two-hybrid selection in yeast. *Genetics*. 144: 1425-1436.

7. Shu T, He X-Y, Chen J-W, Mao Y-S and Gao X-D (2021) The pH-responsive transcription factors YlRim101 and Mhy1 regulate alkaline pH-induced filamentation in the dimorphic yeast *Yarrowia lipolytica*. *mSphere*. 6: e00179-21.

8. Wu H, Shu T, Mao Y-S and Gao X-D (2020) Characterization of the promoter, downstream target genes and recognition DNA sequenced of Mhy1, a key filamentation-promoting transcription factor in the dimorphic yeast *Yarrowia lipolytica*. *Curr. Genet*. 66: 245-261.

Table S4. Oligonucleotides used in this study.

Name	Sequence (5’→3’)	Use
YlURA3-F	CGCTCTAGAACTAGTGGATC	Amplification of Yl*URA3*
YlURA3-R	GACGGTATCGATAAGCTTAT	Amplification of Yl*URA3*
YlLEU2-F	ACCCTTGACGATCTCGTATGTCCC	Amplification of Yl*LEU2*
YlLEU2-R	CCTTCCTTTAATAAACCGACTACAC	Amplification of Yl*LEU2*
CKR	AACATCCAGAGAAGCACACAGG	Checking gene deletion
CKF	CTGTGTATCCGCATGATCTGTC	Checking gene deletion
YlAAF1-PF	GTCTGGCCAGTGCCATACATACC	Amplification of P_{YlAAF1}
YlAAF1-PR	gatccactagttctagagcgGGTTGGCTGTGTGTGCGAAAG	Amplification of P_{YlAAF1}
YlAAF1-TF	ataagcttatcgataccgtCGTGCGGGGGCGTAATTCGTTC	Amplification of T_{YlAAF1}
YlAAF1-TR	catacgagatcgtcaagggTGGAGATGGAGCGTAGGGTTCG	Amplification of T_{YlAAF1}
YlAAF1-5CK	CGGGTTTCCACGTCACATTG	Checking Yl*aaf1*Δ deletion
YlAAF1-3CK	GGCTGTCATTACACTGAGCTC	Checking Yl*aaf1*Δ deletion
YlAAF2-PF	GACCCAGGTGGGCATTGGTTG	Amplification of P_{YlAAF2}
YlAAF2-PR	gatccactagttctagagcGGGGCTGTCTGTGTGCGAGTATC	Amplification of P_{YlAAF2}
YlAAF2- TF	ataagcttatcgataccgtcGGTAGCAATGGTCCACACCTC	Amplification of T_{YlAAF2}
YlAAF2- TR	catacgagatcgtcaagggtTCTGTTGGCGCCAGGAGTG	Amplification of T_{YlAAF2}
YlAAF2-5CK	GTGTGTGTCTGTGGTGTGCC	Checking Yl*aaf2*Δ deletion
YlAAF2-3CK	GCGAGGGAGCACATGACGTTG	Checking Yl*aaf2*Δ deletion
PYlAAF1-HBF	gtttgacagcttatcatcgatTATTTTTTAAATAGTATTTTTAGGTCGATTAGT	Amplification of Yl*AAF1* promoter for compensation
PYlAAF1-HBR	accgcattaaagcttatcgatGGTTGGCTGTGTGTGCGAA	Amplification of Yl*AAF1* promoter for compensation
YlAAF1-HBF	cagcttatcatcgataagcttATGCACCCATACTACCCTCCC	Amplification of Yl*AAF1* ORF for compensation
YlAAF1-HBR	taaactaccgcattaaagcttCGAAGGAAACAACCACATCACTC	Amplification of Yl*AAF1* ORF for compensation
PYlAAF2-HBF	gtttgacagcttatcatcgatTTGTTGAGTTTGGTTGTTACGGTAT	Amplification of Yl*AAF2* promoter for compensation
PYlAAF2-HBR	accgcattaaagcttatcgatGGGGCTGTCTGTGTGCGA	Amplification of Yl*AAF2* promoter for compensation
YlAAF2- HBF	cagcttatcatcgataagcttATGAACAACAACAGCCGGATAAT	Amplification of Yl*AAF2* ORF for compensation
YlAAF2- HBR	taaactaccgcattaaagcttGTAAACGGAAGAGTAGCTTGGGG	Amplification of Yl*AAF2* ORF for compensation
YlAAF1-OEF	caggaattcgatatcaagcttATGCACCCATACTACCCTCCC	Amplification of Yl*AAF1* ORF for overexpression
YlAAF1-OER	gtcgacggtatcgataagcttTTAGACCGCAGTTCCTGGACTAG	Amplification of Yl*AAF1* ORF for overexpression
YlAAF2-OEF	caggaattcgatatcaagcttATGAACAACAACAGCCGGATAAT	Amplification of Yl*AAF2* ORF for overexpression
YlAAF2-OER	gtcgacggtatcgataagcttCGCATCTTTAGTTTGCAGATTG	Amplification of Yl*AAF2*

	A	ORF for overexpression
YlAAF1-DOEF	agaactagtggatcccccgggATGCACCCATACTACCCTCCC	Amplification of Yl*AAF1* ORF for double overexpression
YlAAF1-DOER	atcgaattcctgcagcccgggTTAGACCGCAGTTCCTGGACTAG	Amplification of Yl*AAF1* ORF for double overexpression
RYlAAF2- DOEF	caggaattcgatatcaagcttCGCATCTTTAGTTTGCAGATTGA	Amplification of reverse P_{YlTEF1}-Yl*AAF2* for double overexpression
RYlTEF1-DOER	gtcgacggtatcgataagcttAGAGACCGGGTTGGCGGC	Amplification of reverse P_{YlTEF1}-Yl*AAF2* for double overexpression
YlAAF1-GF	agatctagaactagtggatccTATTTTTTAAATAGTATTTTTAGGTCGATTAGT	Amplification of P_{YlAAF1}-Yl*AAF1* for fluorescence
YlAAF1-GR	accgtcgacaagcttggatccTCGCCTGAAAGCATACGCC	Amplification of P_{YlAAF1}-Yl*AAF1* for fluorescence
YlAAF2-GF	agatctagaactagtggatccTTGTTGAGTTTGGTTGTTACGGTAT	Amplification of P_{YlAAF2}-Yl*AAF2* for fluorescence
YlAAF2-GR	accgtcgacaagcttggatccTTCAACACAATTCCAGCCATTATC	Amplification of P_{YlAAF2}-Yl*AAF2* for fluorescence
YlAAF1-BCF	tcccccgggctgcaggaattcATGCACCCATACTACCCTCCC	Amplification of Yl*AAF1* ORF for BiFC
YlAAF1-BCR	gataagcttgatatcgaattcTCATCGCCTGAAAGCATACGC	Amplification of Yl*AAF1* ORF for BiFC
YlAAF2-BCF	tcccccgggctgcaggaattcATGAACAACAACAGCCGGATAAT	Amplification of Yl*AAF2* ORF for BiFC
YlAAF2-BCR	gataagcttgatatcgaattcTTATTCAACACAATTCCAGCCATT	Amplification of Yl*AAF2* ORF for BiFC
YlAAF1-Y2HF	gaattccccgggggatccATGCACCCATACTACCCTCCC	Amplification of Yl*AAF1* ORF for Y2H
YlAAF1-Y2HR	caggtcgacatcgatggatccTCATCGCCTGAAAGCATACGC	Amplification of Yl*AAF1* ORF for Y2H
YlAAF1S1-Y2HF	gaattccccgggggatccAACTCGGACCGTCCCAAAA	Amplification of Yl*AAF1*$^{126-185}$ for Y2H
YlAAF1S1-Y2HR	caggtcgacatcgatggatccTCAGACCGATCCGGAGCC	Amplification of Yl*AAF1*$^{126-185}$ for Y2H
YlAAF1S2-Y2HF	gaattccccgggggatccGGCCAACAAGGCTCCGGA	Amplification of Yl*AAF1*$^{178-243}$ for Y2H
YlAAF1S2-Y2HR	caggtcgacatcgatggatccTCACTTGAACAGAATGCTGTTTTTG	Amplification of Yl*AAF1*$^{178-243}$ for Y2H
YlAAF2-Y2HF	gaattccccgggggatccATGAACAACAACAGCCGGATAAT	Amplification of Yl*AAF2* ORF for Y2H
YlAAF2-Y2HR	caggtcgacatcgatggatccTTATTCAACACAATTCCAGCCATT	Amplification of Yl*AAF2* ORF for Y2H
YlAAF2S1-Y2HF	gaattccccgggggatccAAAAAGGCCCGAAGAGCCT	Amplification of

		Yl*AAF2*$^{157\text{-}216}$ for Y2H
YlAAF2S1-Y2HR	caggtcgacatcgatggatccTTAGATGAGGTTGCCATTCATCTG	Amplification of Yl*AAF2*$^{157\text{-}216}$ for Y2H
YlAAF2S2-Y2HF	gaattccccgggggatccCTCATGGCCCAGATGAATGG	Amplification of Yl*AAF2*$^{207\text{-}277}$ for Y2H
YlAAF2S2-Y2HR	caggtcgacatcgatggatccTTAGGAGGAGCCTAGGAGCGA	Amplification of Yl*AAF2*$^{207\text{-}277}$ for Y2H
GST-F	gagggaaggatttcagaattcATGTCCCCTATACTAGGTTATTGG	Amplification of *GST* for pull-down
GST-OLF	ATGTCCCCTATACTAGGTTATTGG	Overlapping PCR for *GST* fusion
GST-R	gactctagaggatccgaattcTCATTTTGGAGGATGGTCGCCAC	Amplification of *GST* for pull-down
YlAAF1-PDF	gagggaaggatttcagaattcATGCACCCATACTACCCTCCC	Amplification of Yl*AAF1* ORF for pull-down
YlAAF1-PDR	ccaataacctagtataggggacatTCGCCTGAAAGCATACGC	Amplification of Yl*AAF1* ORF for pull-down
YlAAF1S2Δ-PDR	TCCGGAGCCTTGTTGGCC	Amplification of Yl*AAF1*$^{184\text{-}237\Delta}$ for pull-down
YlAAF1S2Δ-PDF	caacaaggctccggAAACAGCATTCTGTTCAAGGTGG	Amplification of Yl*AAF1*$^{184\text{-}237\Delta}$ for pull-down
YlAAF1S2-PDF	gagggaaggatttcagaattcATGGGCCAACAAGGCTCCGGA	Amplification of Yl*AAF1*$^{178\text{-}243}$ for pull-down
YlAAF1S2-PDR	ccaataacctagtataggggacatCTTGAACAGAATGCTGTTTTTG	Amplification of Yl*AAF1*$^{178\text{-}243}$ for pull-down
YlAAF1N-PDR	ccaataacctagtataggggacaTGGCCGACCGCATCACC	Amplification of Yl*AAF1*$^{1\text{-}125}$ for pull-down
YlAAF1S1-PDF	gagggaaggatttcagaattcATGAACTCGGACCGTCCCAAAA	Amplification of Yl*AAF1*$^{126\text{-}185}$ for pull-down
YlAAF1S1-PDR	ccaataacctagtataggggacatGACCGATCCGGAGCCTTG	Amplification of Yl*AAF1*$^{126\text{-}185}$ for pull-down
YlAAF1C-PDF	gagggaaggatttcagaattcATGGTGGTGTTTGAAAACCGCG	Amplification of Yl*AAF1*$^{244\text{-}355}$ for pull-down
YlAAF2-PDF	gagggaaggatttcagaattcATGAACAACAACAGCCGGATAAT	Amplification of Yl*AAF2* ORF for pull-down
YlAAF2-PDR	ccaataacctagtataggggacatTTCAACACAATTCCAGCCAT	Amplification of Yl*AAF2* ORF for pull-down
YlAAF2S2Δ-PDR	ATTCATCTGGGCCATGAGC	Amplification of Yl*AAF2*$^{213\text{-}267\Delta}$ for pull-down
YlAAF2S2Δ-PDF	gctcatggcccagatgaatGCCGAAAACTCGTCGCTCC	Amplification of Yl*AAF2*$^{213\text{-}267\Delta}$ for pull-down
YlAAF2S2-PDF	gagggaaggatttcagaattcATGCTCATGGCCCAGATGAAT	Amplification of

	GG	Yl*AAF2*$^{207-277}$ for pull-down
YlAAF2S2-PDR	ccaataacctagtataggggacatGGAGGAGCCTAGGAGCGA	Amplification of Yl*AAF2*$^{207-277}$ for pull-down
FLAG-1F	gcgtatgctttcaggcgaGACTACAAAGACCATGACGGTG	Overlapping PCR fusion of Yl*AAF1* and *FLAG* for pull-down
FLAG-2F	atggctggaattgtgttgaaGACTACAAAGACCATGACGGTG	Overlapping PCR fusion of Yl*AAF2* and *FLAG* for pull-down
FLAG-R	gactctagaggatccgaattcCTACTTGTCATCGTCATCCTTGT	Amplification of *FLAG* for pull-down
YlAAF1-FR	TCGCCTGAAAGCATACGC	Overlapping PCR fusion of Yl*AAF1* and *FLAG* for pull-down
YlAAF2-FR	TTCAACACAATTCCAGCCAT	Overlapping PCR fusion of Yl*AAF2* and *FLAG* for pull-down
LEXA-F	caggaattcgatatcaagcttATGAAAGCGTTAACGGCCAG	Amplification of *lexA*
LEXA-R	gtcgacggtatcgataagcttCTATGGTTCACCGGCAGCC	Amplification of *lexA*
LEXA-OLR	TGGTTCACCGGCAGCCAC	Overlapping PCR for *lexA* fusion
YlAAF1-Y1HF	gtggctgccggtgaaccaATGCACCCATACTACCCTCC	Amplification of Yl*AAF1* ORF for Y1H
YlAAF1-Y1HR	gtcgacggtatcgataagcttTCATCGCCTGAAAGCATACGC	Amplification of Yl*AAF1* ORF for Y1H
YlAAF1C-Y1HF	gtggctgccggtgaaccaGTGGTGTTTGAAAACCGCGA	Amplification of Yl*AAF1*$^{244-355}$ for Y1H
YlAAF2-Y1HF	gtggctgccggtgaaccaATGAACAACAACAGCCGGAT	Amplification of Yl*AAF2* ORF for Y1H
YlAAF2-Y1HR	gtcgacggtatcgataagcttTTATTCAACACAATTCCAGCCATT	Amplification of Yl*AAF2* ORF for Y1H
YlAAF1-QF	TGGTGTTTGAAAACCGCGAC	qPCR for Yl*AAF1*
YlAAF1-QR	AGTAGGGCTGAGCGTAAGGA	qPCR for Yl*AAF1*
YlAAF2-QF	AGCCGAAAGCTCGACAAGAA	qPCR for Yl*AAF2*
YlAAF2-QR	GCCTAGGAGCGACGAGTTTT	qPCR for Yl*AAF2*
GAPDH-QF	CCGAGTCCCTACCGTTGATG	qPCR for *GAPDH*
GAPDH-QR	TCATGGTGGCCTTGATGTCC	qPCR for *GAPDH*
YlAAF1-OF	GCGACTTCACAAACACGACGAC	Overlapping PCR fusion of Yl*AAF1* and *FLAG* for WB
YlAAF1-OR	ccgtcatggtctttgtagtcTCGCCTGAAAGCATACGCC	Overlapping PCR fusion of Yl*AAF1* and *FLAG* for WB
YlAAF2-OF	GCTCCATCTTTTGTCTGCTAGCC	Overlapping PCR fusion of Yl*AAF2* and *FLAG* for WB
YlAAF2-OR	ccgtcatggtctttgtagtcTTCAACACAATTCCAGCCATTATC	Overlapping PCR fusion of Yl*AAF2* and *FLAG* for WB

FLAG-OF	GACTACAAAGACCATGACGG	Overlapping PCR for *FLAG* fusion
FLAG-OR	gatccactagttctagagcgctaCTTGTCATCGTCATCCT	Overlapping PCR for *FLAG* fusion
PYlAAF1-F	agaactagtggatccaagcttTATTTTTAAATAGTATTTTTAG GTCGATTAGT	Amplification of Yl*AAF1* promoter for *lacZ*
PYlAAF1-R	cagggtaccgtcgacaagcttCATGGTTGGCTGTGTGTGCG	Amplification of Yl*AAF1* promoter for *lacZ*
PYlAAF2-F	agaactagtggatccaagcttTTGTTGAGTTTGGTTGTTACG GTAT	Amplification of Yl*AAF2* promoter for *lacZ*
PYlAAF2-R	cagggtaccgtcgacaagcttCATGGGGCTGTCTGTGTGCG	Amplification of Yl*AAF2* promoter for *lacZ*
YlAAF1-EMSR	gactctagaggatccgaattcTCATCGCCTGAAAGCATACGC	Amplification of Yl*AAF1* ORF for EMSA
YlAAF2-EMSR	gactctagaggatccgaattcTTATTCAACACAATTCCAGCC ATT	Amplification of Yl*AAF2* ORF for EMSA
YlAAF1-STRE1-F	GCCACCGTTCTGACAACC	Amplification of STRE1 in Yl*AAF1* promoter
YlAAF1-STRE1-R	TTCCGTCTGATATCGTATCTG	Amplification of STRE1 in Yl*AAF1* promoter
YlAAF1-STRE1m-R	GAGTGTCcGGGGTGGCAGGTAGGTAAG	Amplification of STRE1m in Yl*AAF1* promoter
YlAAF1-STRE1m-F	TGCCACCCCgGACACTCACATACACTGTACG	Amplification of STRE1m in Yl*AAF1* promoter
YlAAF1-STRE2-F	ACCGAACCTTGGAGCAGAC	Amplification of STRE2 in Yl*AAF1* promoter
YlAAF1-STRE2-R	GTCTGGCAATTTCAACTCC	Amplification of STRE2 in Yl*AAF1* promoter
YlAAF1-STRE2m-R	TAGATCCCCgATTTCATGCACCTTGTCTGC	Amplification of STRE2m in Yl*AAF1* promoter
YlAAF1-STRE2m-F	ATGAAATcGGGGATCTAACATTCACCATAC	Amplification of STRE2m in Yl*AAF1* promoter
YlAAF1-STRE3-F	CTTCTTTCTCAGCACCACTTC	Amplification of STRE3 in Yl*AAF1* promoter
YlAAF1-STRE3-R	TTCATGCACCTTGTCTGC	Amplification of STRE3 in Yl*AAF1* promoter
YlAAF1-STRE3m-R	TCGGTTTcGGGGGAGCGAGAGGCGG	Amplification of STRE3m in Yl*AAF1* promoter
YlAAF1-STRE3m-F	CGCTCCCCCgAAACCGAACCTTGGAGCAG	Amplification of STRE3m in Yl*AAF1* promoter
YlAAF2-STRE1-F	GTGTTGGCCACCAACGTC	Amplification of STRE1 in Yl*AAF2* promoter
YlAAF2-STRE1-R	TGCCAAATTACACACACACACACACACAAG	Amplification of STRE1 in Yl*AAF2* promoter
YlAAF2-STRE1m-R	GTCAACCCCgAATGGTGTCTTCACTTCATTC	Amplification of STRE1m

		in Yl*AAF2* promoter
YlAAF2-STRE1m-F	CACCATTcGGGGTTGACTCTGTCGTG	Amplification of STRE1m in Yl*AAF2* promoter
YlAAF2-STRE2-F	ATCACGCGTGAGATGATG	Amplification of STRE2 in Yl*AAF2* promoter
YlAAF2-STRE2-R	CAACATTGCAGCCACACTTG	Amplification of STRE2 in Yl*AAF2* promoter
YlAAF2-STRE2m-R	CCCGCCCCgCACGGCCTTCTGTGTCTC	Amplification of STRE2m in Yl*AAF2* promoter
YlAAF2-STRE2m-F	GCCGTGcGGGGCGGGGCTTGCTGGC	Amplification of STRE2m in Yl*AAF2* promoter
YlAAF2-STRE4/3-F	TGCAATAACAGACCTACCCG	Amplification of STRE4/3 in Yl*AAF2* promoter
YlAAF2-STRE4/3-R	ACCCACAGCAGCTATTATTAG	Amplification of STRE4/3 in Yl*AAF2* promoter
YlAAF2-STRE3m-R	TGTTACCCCgGACGGTGATGACTTGCCC	Amplification of STRE3m in Yl*AAF2* promoter
YlAAF2-STRE3m-F	CACCGTCcGGGGTAACATTGAGGGCAAG	Amplification of STRE3m in Yl*AAF2* promoter
YlAAF2-STRE4m-R	ACTTGCCCCgGTGGCGTATAACACTGGAC	Amplification of STRE4m in Yl*AAF2* promoter
YlAAF2-STRE4m-F	ACGCCACcGGGGCAAGTCATCACCGTC	Amplification of STRE4m in Yl*AAF2* promoter
YlAAF2-STRE4m/3m-R	CCCCgGACGGTGATGACTTGCCCCgGTGGCGTATAACACTGGAC	Amplification of STRE4m/3m in Yl*AAF2* promoter
YlAAF2-STRE4m/3m-F	ACcGGGGCAAGTCATCACCGTCcGGGGTAACATTGAGGGCAAG	Amplification of STRE4m/3m in Yl*AAF2* promoter
PMHY1-775-F	ACCTCCCTTCGTGGTTTATG	Amplification of 150-bp probe in *MHY1* promoter
PMHY1-626-R	ATTGACTTCGGCCAGACTTTTC	Amplification of 150-bp probe in *MHY1* promoter

Note: Gene sequences are written in capital letters. Restriction sites used for cloning are underlined.
